# The Effect of Thermal Stress on the Physiology and Bacterial Communities of Two Key Mediterranean Gorgonians

**DOI:** 10.1128/aem.02340-21

**Published:** 2022-03-22

**Authors:** Romie Tignat-Perrier, Jeroen A. J. M. van de Water, Dorian Guillemain, Didier Aurelle, Denis Allemand, Christine Ferrier-Pagès

**Affiliations:** a Unité de Recherche sur la Biologie des Coraux Précieux CSM - CHANEL, Centre Scientifique de Monaco, Monaco, Principality of Monaco; b Coral Ecophysiology Laboratory, Centre Scientifique de Monaco, Monaco, Principality of Monaco; c Centre Scientifique de Monaco, Monaco, Principality of Monaco; d Aix Marseille Université, CNRS, IRD, IRSTEA, OSU Institut Pythéas, Marseille, France; e Aix Marseille Université, Université de Toulon, CNRS, IRD, MIO, Marseille, France; f Institut de Systématique, Evolution, Biodiversité (ISYEB), Muséum national d'Histoire naturelle, CNRS, Sorbonne Université, EPHE, Université des Antilles, Paris, France; Royal Netherlands Institute for Sea Research

**Keywords:** antioxidant, coral, gorgonian, microbiota, octocoral, physiology, thermal stress, upwelling

## Abstract

Gorgonians are important habitat-providing species in the Mediterranean Sea, but their populations are declining due to microbial diseases and repeated mass mortality events caused by summer heat waves. Elevated seawater temperatures may impact the stress tolerance and disease resistance of gorgonians and lead to disturbances in their microbiota. However, our knowledge of the biological response of the gorgonian holobiont (*i.e.,* the host and its microbiota) to thermal stress remains limited. Here, we investigated how the holobiont of two gorgonian species (*Paramuricea clavata* and *Eunicella cavolini*) are affected throughout a 7-week thermal stress event by following both the corals’ physiology and the composition of their bacterial communities. We found that *P. clavata* was more sensitive to elevated seawater temperatures than *E. cavolini*, showing a greater loss in energy reserves, reduced feeding ability, and partial mortality. This lower thermotolerance may be linked to the ∼20× lower antioxidant defense capacity in *P. clavata* compared with *E. cavolini*. In the first 4 weeks of thermal stress, we also observed minor shifts in the microbiota of both species, suggesting that the microbiota likely plays a limited role in thermal acclimation of the holobiont. However, major stochastic changes occurred later on in some colonies, which were of a transient nature in *E. cavolini*, but were linked to partial colony mortality in *P. clavata*. Overall, our results show significant, but differential, effects of thermal stress on the holobionts of both *E. cavolini* and *P. clavata* and predict potentially severe impacts on gorgonian populations under future climate scenarios.

**IMPORTANCE** In the Mediterranean Sea, the tree-shaped gorgonian corals form large forests that provide a place to live for many species. Because of this important ecological role, it is crucial to understand how common habitat-forming gorgonians, like *Eunicella cavolini* and *Paramuricea clavata*, are affected by high seawater temperatures that are expected in the future due to climate change. We found that both species lost biomass, but *P. clavata* was more affected, being also unable to feed and showing signs of mortality. The microbiota of both gorgonians also changed substantively under high temperatures. Although this could be linked to partial colony mortality in *P. clavata*, the changes were temporary in *E. cavolini.* The overall higher resistance of *E. cavolini* may be related to its much higher antioxidant defense levels than *P. clavata.* Climate change may thus have severe impacts on gorgonian populations and the habitats they provide.

## INTRODUCTION

The Mediterranean Sea is one of the regions in the world most vulnerable to seawater warming due to its small size and enclosed configuration (1–3). It is therefore a relevant study area to investigate the impacts of climate change on marine benthic organisms, such as gorgonians. These corals (Octocorallia) can form dense “animal forests” thereby providing critical habitat for other species on the typical Mediterranean coralligenous habitats. Over the last decades, shallow populations of gorgonians have already been impacted severely (4, 5) due to microbial diseases and mass mortality events caused by climatic anomalies in summer (6, 7). In fact, two types of thermal anomalies can occur: heatwaves (i.e., up to 27°C during a few days) or long periods of above-threshold temperatures (i.e., >24°C) that may be interrupted by upwelling events of dense and cold water coming from the deep sea (7, 8). During an upwelling, the temperature of the top 50 m of the water column can decrease by 10°C within a few hours, and then gradually increase back to high temperatures within a few days (9). While these upwelling events induce large and fast temperature fluctuations potentially inducing additional stress to gorgonians, they may also bring short-term relief.

The biological response of gorgonians to thermal stress is currently not well understood, and the effects of upwelling events on the gorgonian physiology have not been studied yet. So far, only short- and long-term continuous exposures to high seawater temperatures have been tested (10–13), and these studies have shown species- and population-dependent responses to thermal stress (14, 15). Under both short- and long-term stress, tissue necrosis was observed in colonies of *Paramuricea clavata* and *Corallium rubrum*, whereas *Eunicella cavolini* and *Eunicella singularis* displayed greater thermotolerance with no obvious signs of necrosis (14–16). However, tissue discoloration and reductions in spicule calcification and lipid energy reserves have been reported for heat-stressed *Eunicella* spp. (12, 15). Differential adaptive or acclimation abilities have been shown to depend on the depth at which coral species live, their thermal history, and their genetic drift (10, 16–20). The antioxidant capacity is an important component of the stress tolerance in anthozoans (21–25), and may be one of the adaptive or acclimation abilities that play a key role in the differential thermotolerance of gorgonians. Levels of cellular reactive oxygen and nitrogen species (ROS and RNS, respectively) often increase under stressful conditions. As ROS and RNS can damage lipids, proteins, and DNA (26, 27), anthozoans produce a wide range of enzymatic and nonenzymatic antioxidant molecules to protect themselves against these damaging impacts (21, 23). However, when the production of ROS and RNS exceeds the antioxidant capacity of an organism, oxidative stress occurs.

Temperature changes may also induce shifts in the gorgonian microbiota. Gorgonians engage in symbiotic relationships with microorganisms, including bacteria, fungi, archaea, and viruses, which together form an entity called a “holobiont” (28). Previous investigations of several Mediterranean gorgonian species highlighted a dominant and species-specific core microbiota, suggesting that the host selects for microorganisms of certain taxa that may be of importance to the overall health of the holobiont (29–31). The most dominant bacterial symbiont is *Endozoicomonas*, which is a widespread symbiont of marine invertebrates and is believed to be involved in nutrient acquisition and provision, and the structuring of the host microbiota and the prevention of pathogen proliferation through the production of quorum-sensing and antimicrobial molecules (32). While the extent of their functions remains to be further investigated, gorgonian-associated microorganisms may also be implicated in the stress tolerance of their host, as has been described for some scleractinian corals (33–35). For example, antioxidant compounds may be produced by some microbial symbionts of corals (35, 36).

Thermal stress may induce changes in the microbiota by exerting a downward pressure on the survival of beneficial microorganisms, which could influence the stress tolerance of gorgonians and thereby promote temperature-dependent bacterial diseases. Shifts in the microbiota of some tropical reef-building coral species have indeed been observed during and after thermal stress that may be used to predict host tolerance (33, 37, 38). On the contrary, the microbiota of other scleractinian coral species have been shown to be stable and inflexible despite changes in the physiological state of the host (39–44). The gorgonian microbiota might contribute to the response of the holobiont to environmental stress and thus impact its stress tolerance and disease resistance. However, no investigations have been conducted on the dynamics of the gorgonian microbiota under thermal stress.

In this study, we investigated how the holobionts of two common Mediterranean gorgonian species (*Eunicella cavolini* and *Paramuricea clavata*) respond to a simulated summer thermal regime with elevated temperatures interrupted by upwelling events. These two species with similar depth distribution ranges (10–100 m) are known to exhibit differential responses to prolonged events of warm temperatures, but are both characterized by a *Endozoicomonas-*dominated microbiota (30). By assessing the dynamics of the gorgonian-associated bacterial community along with the hosts’ physiological state, including total antioxidant capacity and energy metabolism, we aimed to evaluate the impact of thermal stress on the gorgonian holobiont and to link host response to changes in microbiota.

## RESULTS

### Host physiological response to thermal stress.

**FIG 1:**
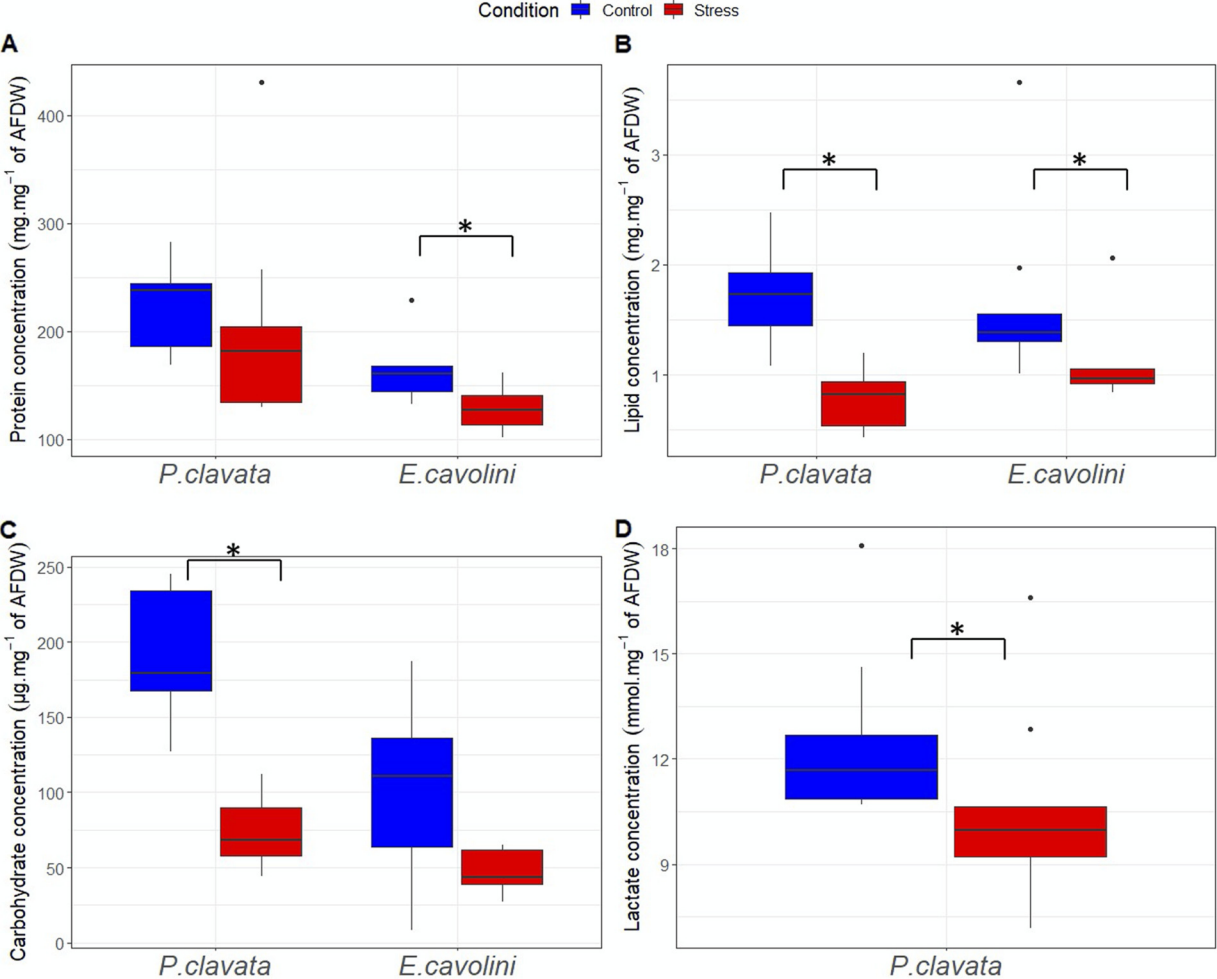
Impact of 56 days of thermal stress on host tissue composition. Protein (A; in mg of proteins per mg of AFDW), lipid (B; in mg of lipids per mg of AFDW), carbohydrate (C; in μg of carbohydrates per mg of AFDW), and lactate (D; in nmol of lactates per mg of AFDW) concentration in the tissues of *E. cavolini* and *P. clavata* under control and thermal stress conditions at T6. Lactate was not detected in *E. cavolini*. Asterisks indicate significant differences (*P* < 0.05) between conditions.

Under control conditions, the protein and carbohydrate contents per mg ash free dry weight (AFDW) were on average higher in *P. clavata* compared with *E. cavolini* (*P* = 2.6 × 10^−3^ and 2.1 × 10^−6^, respectively). *E. cavolini* exposed for 7 weeks to thermal stress had 20% lower protein (*P* = 5.2 × 10^−3^) and 30% lower lipid (*P* = 1.0 × 10^−2^) reserves than under control temperature conditions. The lipid and carbohydrate reserves of *P. clavata* were 55% (*P* = 2.1 × 10^−5^) and 58% (*P* = 2.0 × 10^−2^) lower in thermally stressed fragments (Fig. 1 and Table S1 in the supplemental material). Lactate concentrations were on average lower in *P. clavata* colonies that experienced thermal stress than those under control conditions (*P* = 1.8 × 10^−4^). Lactate was not detectable in *E. cavolini*.

From time point T4 onwards, polyp inactivity (i.e., absence of a response from po-lyps to food supply) was visible on all stressed *P. clavata* fragments, and tissue loss was visible on colonies 1, 3, and 6 (see pictures in Fig. S2), while no visual impacts were observed on fragments under control conditions. *E. cavolini* did not show any sign of polyp inactivity and tissue loss in both conditions.

**FIG 2:**
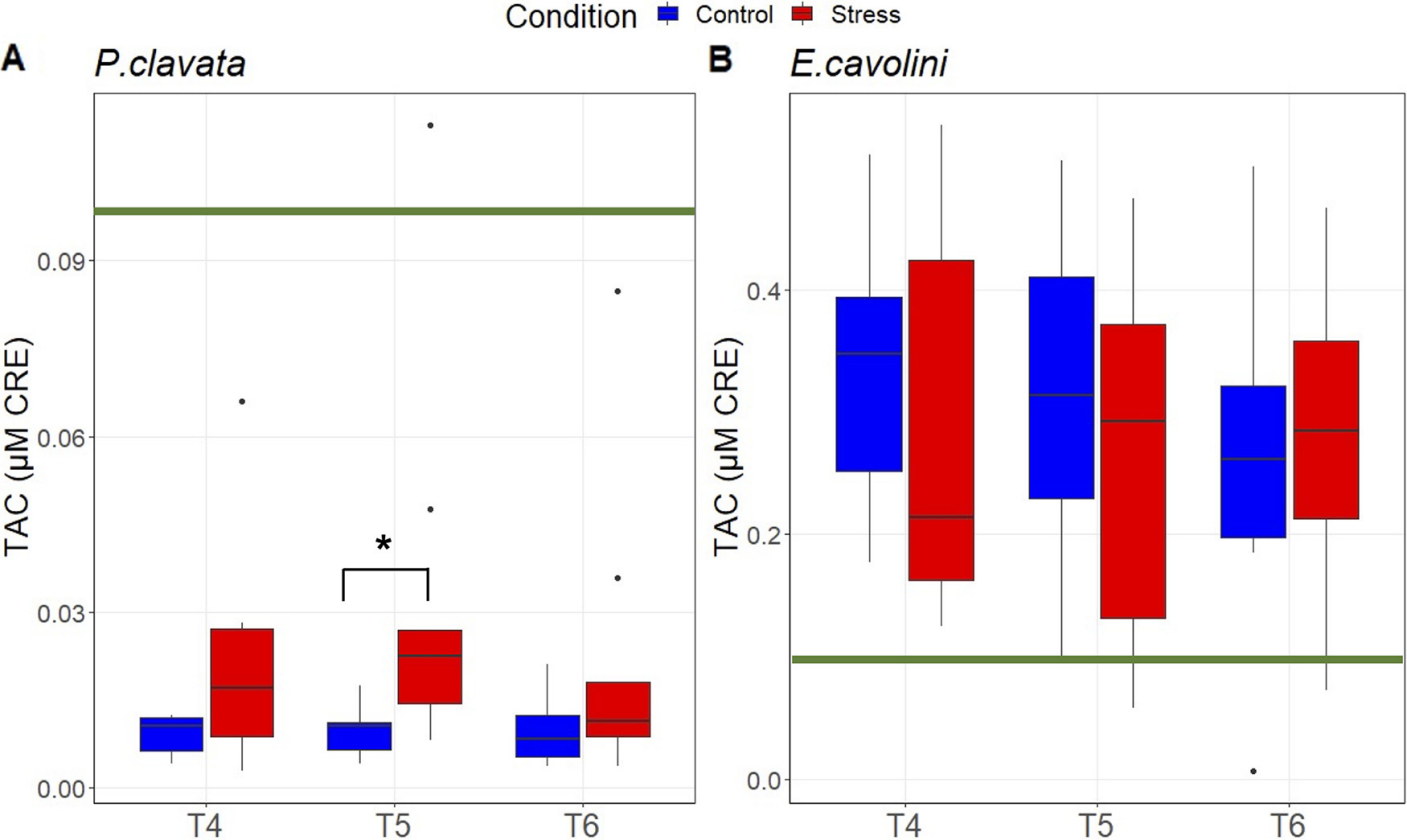
Total antioxidant capacity of gorgonians during the second simulated upwelling event. Total antioxidant capacity in μM of CRE (copper reducing equivalents) per mg of AFDW of *P. clavata* (A) and *E. cavolini* (B) under control and thermal stress conditions before (T4), during (T5), and after (T6) the second simulated upwelling event. Asterisks indicate significant differences (*P* < 0.05) between conditions. The green bar is at 0.1 μM CRE in both graphs, emphasizing the interspecies differences.

Total antioxidant capacity (TAC) per mg AFDW did not increase during thermal stress (at T4, T5, and T6) in both *E. cavolini* and *P. clavata* (Fig. 2). However, the intercolony variance was large in both treatments. Overall, *E. cavolini* had ∼20 times higher TAC compared with *P. clavata* (*P* = 2.3 × 10^−6^).

### Changes in bacterial community composition during thermal stress.

Chao1 estimated richness was on average higher for *E. cavolini* (143 ± 62 operational taxonomic units [OTUs] on average based on the control colonies) than for *P. clavata* (115 ± 40 OTUs; *P* = 3.8 × 10^−4^; Table S1 and S2). *E. cavolini* colonies that were exposed to thermal stress conditions showed on average higher Chao1 richness estimations (246 ± 154) compared with the same colonies under control conditions (144 ± 63) (*P* = 1.5 × 10^−6^). For *P. clavata*, higher Chao1 estimations were also observed in the co-lonies under stress conditions compared with the colonies under control conditions at T4 and T6 (*P* = 5.2 × 10^−3^ and 3.0 × 10^−4^, respectively).

**FIG 3:**
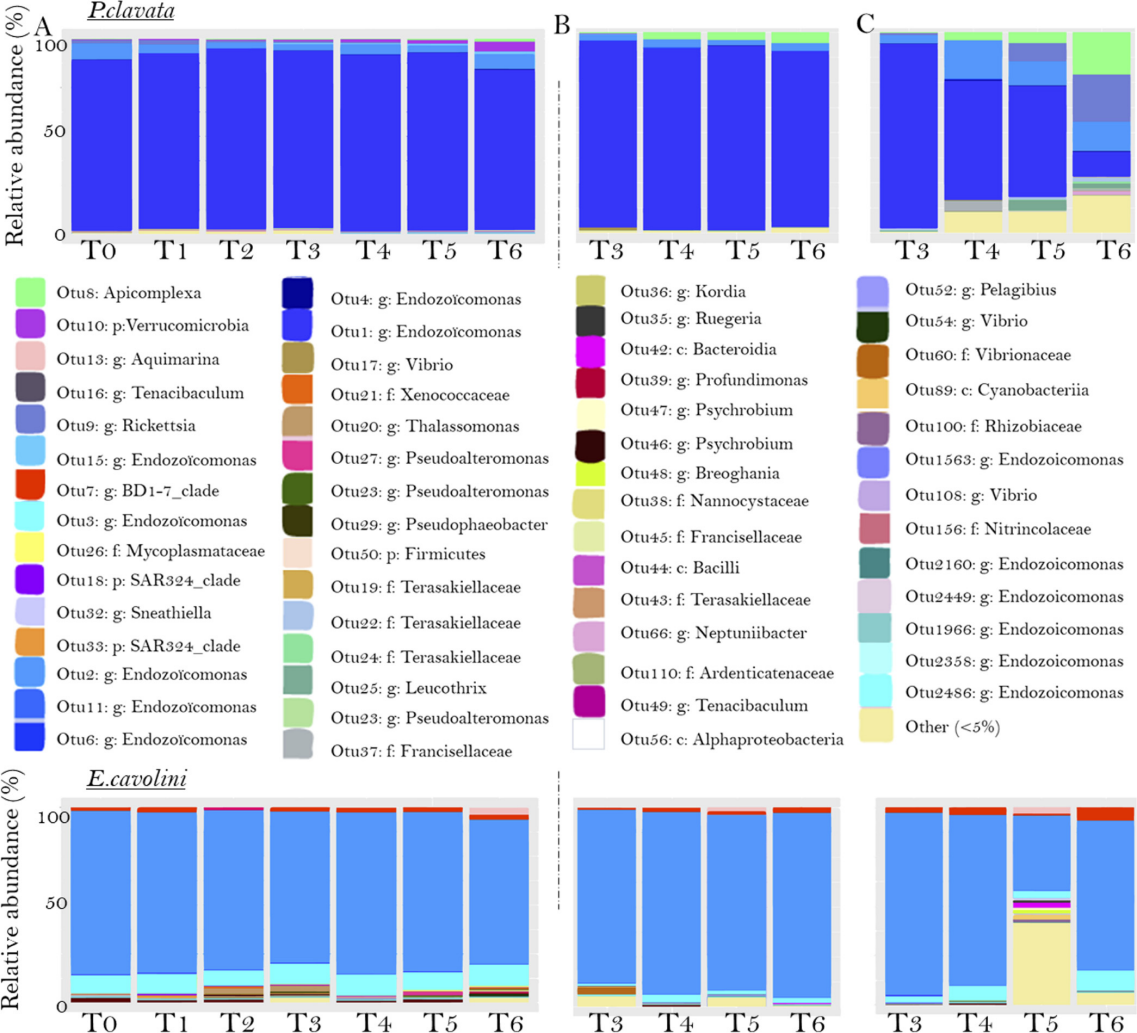
Relative abundance of the most abundant bacterial OTUs over time. Structure of the bacterial communities associated with the control colonies (A; relative composition averaged over the control colonies); one representative colony that was exposed to the thermal stress condition and showed minor changes in its bacterial community (B; colony 7 for both *P. clavata* and *E. cavolini* belonging to the A group), and one representative a colony that was exposed to the thermal stress condition and showed large changes in its bacterial community (C; colony 1 for both *P. clavata* and *E. cavolini* belonging to the B group).

The bacterial communities of both *E. cavolini* and *P. clavata* were dominated by *Endozoicomonas* bacteria under control conditions, although they were characterized by different OTUs of *Endozoicomonas* (Fig. 3 and Fig. S3). The most abundant OTUs were OTU1-*Endozoicomonas* (89.2%), OTU2-*Endozoicomonas* (4.8%), OTU10-*Verrucomicrobia* (1.6%), OTU9-*Rickettsia* (0.80%), and OTU8-*Apicomplexa* (0.62%) for *P. clavata*, whereas OTU2-*Endozoicomonas* (80.2%), OTU3-*Endozoicomonas* (9.9%), OTU7-*BD1-7 clade* (2.2%), OTU20-*Thalassomonas* (0.85%), and OTU13-*Aquimarina* (0.64%) were dominating the microbiota of *E. cavolini*. However, the relative abundance of these OTUs varied depending on time and colony (Fig. S3).

**FIG 4:**
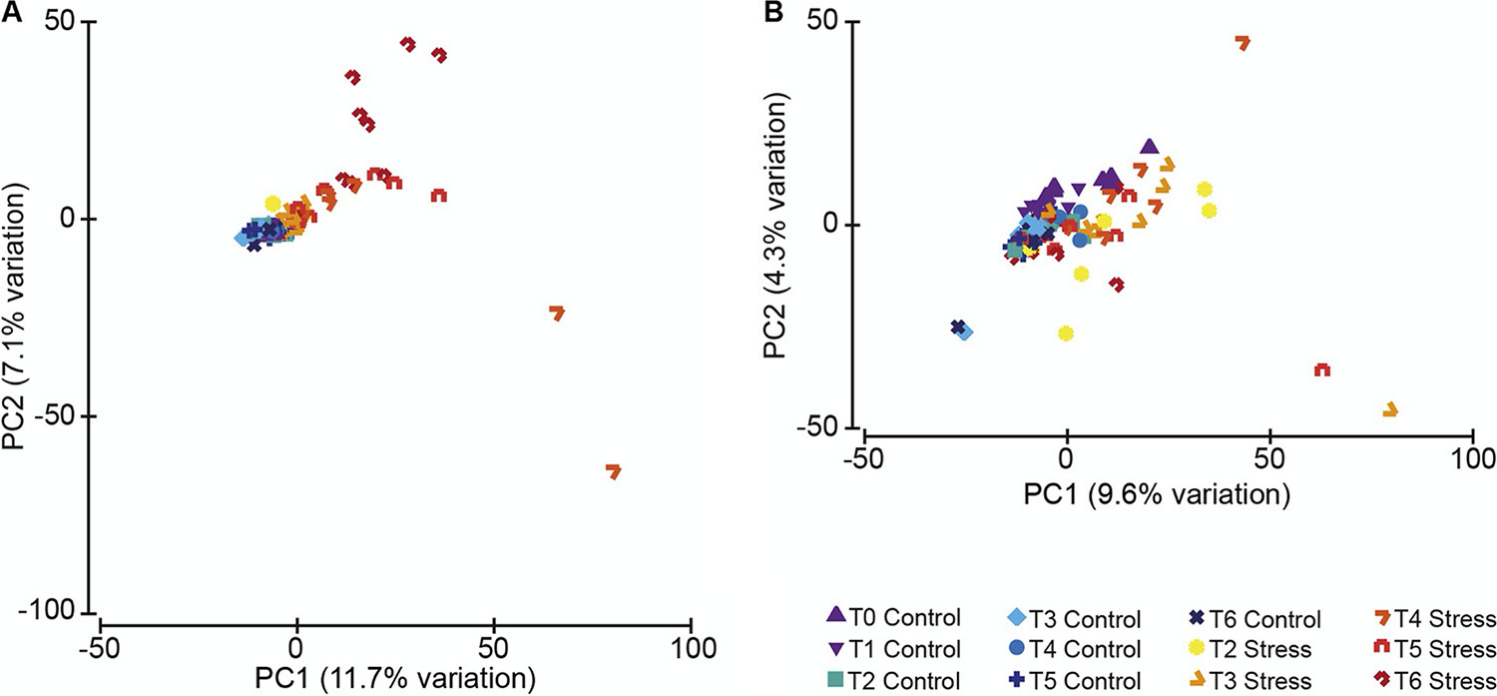
Beta diversity of the bacterial community of the gorgonians under control and thermal stress conditions. Principal component analysis based on the Aitchison distance matrix showing the distribution and dispersion of the samples (A: *P. clavata*; B: *E. cavolini*).

Beta diversity analyses of the bacterial communities showed that the microbiota of colonies of *P. clavata* under control conditions were similar, grouping tightly together, and were significantly different from the microbiota of colonies exposed to thermal stress conditions (*P* = 2.9 × 10^−3^), whose dispersion was also significantly higher (*P* = 1 × 10^−4^; Fig. 4A; statistical results Table S3). Besides, differences between time points were observed (*P* = 1 × 10^−4^), but there was significant variability among colonies (*P* = 1 × 10^−4^). Similar patterns were observed in *E. cavolini* (thermal condition *P* = 3.9 × 10^−3^; time point *P* = 1 × 10^−4^; dispersion: *P* = 6 × 10^−4^), with significant variability between colonies (*P* = 1 × 10^−4^) (Fig. 4B; statistical results Table S3).

Given the significant variability among colonies, hierarchical cluster analyses were performed, which revealed that the high level of dispersion among samples from thermal stress conditions was driven by samples from a few colonies (Fig. S4). These results were then used to objectively assign colonies to either (i) the A group containing colonies whose microbiota showed major significant changes under thermal stress or (ii) the B group containing colonies whose microbiota changed to a small but still significant extent under thermal stress. Using this analysis and the definition that at least two samples from different time points (T4, T5, and/or T6) from the same colony should fall outside of the main cluster of the clustering tree (Fig. S4) to be considered “large change,” colonies 1, 3, 6, and 9 of *P. clavata* and colonies 1, 5, 6, and 9 of *E. cavolini* were assigned to the A group.

Taking into account these two groups, the effect of thermal condition and time points were assessed (statistical results Table S4). Changes in the microbiota appeared in both species after time point T2, i.e., after prolonged thermal stress and not immediately after the temperature increase. At T3, thermal stress had a small but significant impact on the microbiota of *P. clavata* (*P* = 7 × 10^−4^) and *E. cavolini* (*P* = 2.3 × 10^−3^), but no differences between the A and B groups were observed yet (Fig. S6A, E). More significant impacts were observed between T4 and T6. Specifically, no temporal changes or group differences were observed in *P. clavata* (Fig. S6B–D) under control conditions during this time period, but the microbiota of their paired fragments exposed to thermal stress were consistently different from controls (all time points *P* < 0.0042) and showed clear group-dependent differences (*P* = 9 × 10^−4^). Under thermal stress, the microbiota of the corals from the A group showed large dispersion at all time points and were significantly different from the B group, which was tightly clustered at T4 (*P* = 0.0171) and T5 (*P* = 0.0087). At T6, however, significant changes in the microbiota of the B group occurred in comparison with T4 (*P* = 0.0678, but large dispersion differences *P* = 0.0071) and T5 (*P* = 0.0229), thereby also increasing the dispersion and overlapping with the A group (*P* = 0.603; dispersion *P* = 0.1323). In *E. cavolini*, no significant differences between the A and B groups were observed, but there were changes in the microbiota over time in both control and thermally stressed corals. In addition, thermal stress had a significant impact on the microbiota of *E. cavolini* (Fig. S6F–I) at all three time points (T4 *P* = 0.0067; T5 *P* = 0.0016; T6 *P* = 0.0013), showing also higher dispersion in the beta diversity of the microbiota of thermally stressed corals (T4 *P* = 0.0294; T5 *P* = 0.0048; except T6 *P* = 0.2165 due to one highly divergent control sample).

Next, differential abundance analyses were performed to assess which OTUs were different between either the A or B group and their respective controls at the three different time points. The differential abundance analyses identified significant differences in the abundance of numerous OTUs between the control and thermal stress conditions (Fig. S7 and S8). In *P. clavata*, most of the OTUs whose relative abundance significantly changed between the control and stressed colonies were common in both A and B groups. However, the relative abundance of some of these OTUs changed more in colonies from the A group. For example, the relative abundance of the *Endozoicomonas* genus was lower in stressed than in control colonies. Still, there was a larger decrease in OTU2160-*Endozoicomonas* in the A group of colonies at T6 (A group: reduced by 67% in stressed colonies; B group: reduced by 28% in stressed colonies; Fig. S7). In *E. cavolini*, the differences in OTU changes between the two groups were less pronounced. Still, the relative abundance of some OTUs such as OTU30-*Ruegeria* increased to a larger extent in colonies from the A group than from the B group (A group: increased by 88% in stressed colonies; B group: increased by 63% in stressed colonies; Fig. S8).

The majority of the OTUs differentially abundant between the control and thermal stress conditions were common to all colonies under thermal stress. However, colony-specific changes were observed for several OTUs, likely underlying the high levels of dispersion observed. For example, only in *P. clavata* colony 1, increases in the relative abundance of OTU9-*Rickettsia* (from 0.2 to 23.5%) and OTU25-*Leucothrix* (from 0.002 to 2.5%) were observed under thermal stress at T6. And in *P. clavata* colony 3, similar observations were made for OTU19-*Terasakiellaceae* (from 0.006 to 18.2%) and OTU24-*Terasakiellaceae* (from 0.002 to 3.5%) (Fig. S3). For *E. cavolini*, thermal stress caused an increase in the relative abundance of OTU13-*Aquimarina* (from 0 to 3.0%) and OTU42-*Bacteroidia* (0 and 2.6%) in colony 1 at T5, whereas OTU7-*BD1-7 clade* (2.0 and 5.1% at T6) and OTU18-*SAR324 clade* (0.071 and 2.6%) increased in colony 6 (Fig. S3).

No link was observed between the changes in the microbiota and the changes in the physiology or TAC of the gorgonians based on dbRDA analyses. However, for *P. clavata*, most of the colonies that showed large changes in their microbiota also showed polyp inactivity and tissue loss (Fig. S2).

## DISCUSSION

### Importance of antioxidants for the thermotolerance of gorgonians.

Results from this study suggest that the antioxidant capacity of each species may be important in gorgonian stress tolerance, as previously observed in other anthozoans (21, 23, 45). Under thermal stress, cellular reactive oxygen and nitrogen species (ROS and RNS) levels often increase and may cause damage to lipids, proteins, and DNA. The production of enzymatic and nonenzymatic antioxidant molecules is crucial to defend against ROS/RNS and prevent oxidative stress-induced damages during thermal stress (21, 23). Our results show a differential antioxidant capacity between the two gorgonian species studied here, with *E. cavolini* having a 20 times higher antioxidant capacity than *P. clavata*. The antioxidant capacity in *P. clavata* did not increase under thermal stress and remained thus likely insufficient to neutralize oxidative stress and prevent tissue damage after 7 weeks of heat stress. It should, however, be noted that as we did not measure ROS and RNS levels but only the antioxidant capacity, it is not certain whether these gorgonians experienced oxidative stress. In *E. cavolini*, the antioxidant capacity did not increase under thermal stress either, but the high basal levels likely prevented excessive oxidative stress and hence tissue necrosis during our experiment. The differential investment in antioxidant defense capacity between *E. cavolini* and *P. clavata* may explain the species-specific impacts of the heat stress on their energy reserves. While *P. clavata* had more energy reserves than *E. cavolini* at the beginning of the experiment, the significant reduction in lipid (58% in *P. clavata* and 30% in *E. cavolini*) and carbohydrate (58% in *P. clavata* and 45% in *E. cavolini*) reserves over time may indicate that *P. clavata* allocated significant energy resources to repair tissue damages likely caused by oxidative stress. In addition, the presence of lactate under control conditions may suggest that *P. clavata* has relatively inefficient energy production mechanisms leading to the buildup of potentially toxic end-products (e.g., lactate). This could have significant impacts when conditions change and an organism must rely on anaerobic ATP generation for survival, as potentially lethal levels of lactate that damage its tissues may be reached. The observed decrease in lactate levels in *P. clavata* under thermal stress may, however, be linked to the dramatic drop in carbohydrate (e.g., glycogen) stores and indicate a depletion of its energy reserves leading to starvation of the tissues that may have already suffered from lactate-induced damage.

Besides, *P. clavata* did not feed due to polyp inactivity shortly after the experimental thermal stress levels were reached, despite an abundance of planktonic prey in the seawater, and was therefore unable to fill up its energy reserves needed to repair its tissues. Overall, this may explain the partial mortality observed in *P. clavata*. In contrast, *E. cavolini* continued to feed throughout the experiment regardless of temperature, and its energy reserves were therefore also less impacted. This may thus be one of the reasons why *E. cavolini* is more thermotolerant than *P. clavata* and did not suffer any tissue loss in our experiment.

The different levels of thermotolerance between gorgonian species and even between individuals has already been shown in the literature, based on measures of necrosis, polyp activity, oxygen consumption and calcification rates. However, these measures were unable to fully identify the mechanisms involved in thermotolerance (14, 16, 17, 46). Depth optimum distribution at which species live, thermal history, and genetic drift have been suggested to play a role in the thermal tolerance of gorgonians (10, 16–20). Although *E. cavolini* and *P. clavata* were collected from the same depth in this study, the observed differences in antioxidant capacity between these two species might be related to their different optimal depth distributions. *E. cavolini* usually lives at shallower depths (10–70 m), where thermal fluctuations are larger than at greater depths where *P. clavata* more commonly lives (35–80 m) (14, 47). In addition, *E. cavolini* has been shown to have a faster coenenchyme regeneration capacity than *P. clavata*, suggesting that *E. cavolini* may be more adapted to rapidly recover from stress-related injuries (14).

Here, we documented significant differences in the impacts of elevated seawater temperatures on the physiology of two gorgonian species, indicating particularly that the basal TAC levels may be a predictor for sensitivity to thermal stress. To further investigate the differences in thermotolerance, it may be interesting to study how energy resources are exactly allocated within these gorgonian holobionts under stressful conditions and which processes may be affected using molecular techniques.

### Colony-dependent changes in the microbiota of both gorgonian species under heat stress.

In our heat stress experiment, the composition of bacterial communities showed minor or major changes depending on the colony in both *P. clavata* and *E. cavolini*. In *P. clavata*, all colonies showed minor changes in the composition of bacterial communities during the first 4 weeks of heat stress, when gorgonian colonies likely mounted a defense against the negative impacts of heat stress and the holobiont tried to acclimate to the new conditions. From time point T4 onwards, however, the composition of bacterial communities showed major changes in a few colonies of *P. clavata* (A group) whose tissues were damaged, which appeared following a prolonged period of reduced feeding and thus likely led to a significant depletion of energy stores under thermal stress. Changes in the microbiota that only occurred when the host is in a poor physiological state have also been observed for coral species whose composition of the bacterial communities shows normally little changes under stressful conditions (48). The reduced energy reserves may have led to a reduced investment in antimicrobial defense and microbiota regulatory capacities by the coral host. Previously, energetic constraints along with elevated temperatures have also been linked to the development of mortality events in *P. clavata* (49). Altogether, these observations suggest that a loss of control of the microbiota by the host may be the underlying cause of the major changes observed in the composition of the bacterial communities. The other colonies of *P. clavata* (B group) showed a delayed (about 1 week) onset of large changes in their microbial community structure and no tissue damage. This shows that they were more tolerant to thermal stress than their conspecifics from the A group, but that ultimately the host is also significantly impacted. As we only assessed the energy reserves at the end of the experiment, it remains to be investigated what may explain the differences between the A and B groups.

In *E. cavolini*, a minor shift in the microbiota also appeared in all colonies under thermal stress, yet transient major changes in the composition of the bacterial communities were observed in a few colonies under both thermal conditions. However, these colonies did not visually appear to be more affected by the heat stress than the others. As these transient changes appeared under both control and thermal stress conditions, they were not linked to the heat stress. While the cause of these shifts remains unknown, they might be rather naturally occurring transient changes within the holobiont of this species.

Both minor and major changes observed in the composition of the bacterial communities of *E. cavolini* and *P. clavata* under heat stress were linked to an increase in bacterial diversity as most frequently observed in the literature (36, 50). Shifts in the microbiota may also be related to the acclimation potential of a coral species. Recently, the term “microbiome flexibility” was coined after identifying coral species that maintained a stable microbiota with its beneficial functions in metabolism and protection regardless of the environmental conditions (microbiome regulators) and those that adapt their microbiota by introducing new microbes with beneficial functions for rapid holobiont acclimation to environmental changes (microbiome conformers) (51). The extent of “microbiome flexibility” is thus highly species-specific. Recently, the gorgonian *E. cavolini* was identified as a “microbiome regulator” (52), which is consistent with our results showing primarily minor changes in the microbiota under thermal stress. This suggests that the native microbiota of these species may provide some beneficial functions for the acclimation to elevated seawater temperatures. To further investigate which microbial functions may contribute to a gorgonian holobiont’s acclimation potential and which microbes are involved as well as the implications of the observed stochastic changes on holobiont functioning, future studies would benefit from functional analyses of the microbiota, including metatranscriptomics in combination with metagenomics analyses (53).

Because of the dominance of *Endozoicomonas* (up to 95%) in the microbiota of both gorgonian species, changes in the bacterial community composition were characterized by a general decrease in the relative abundance of *Endozoicomonas* bacteria. Given their importance in coral health through provision of nutrients and microbiota-structuring properties, such decreases in the abundance of *Endozoicomonas* may have adverse implications for holobiont health.

Consequently, an increase in the relative abundances of various other bacterial taxa was observed. The role of these bacteria is, however, unknown as it was not possible to identify the species using the present methodology. Although functions may be inferred, the bacteria that increased in abundance under thermal stress belonged to genera such as *Vibrio*, *Pseudoalteromonas*, and *Ruegeria*, which have previously been implicated in disease (54–56) but have also been considered putative symbionts of corals (30, 57, 58). Metagenomics approaches will allow us to further investigate the functional traits of the bacteria whose relative abundance changed under thermal stress and formulate hypotheses on their specific roles in the holobiont response to thermal stress or whether they are pathogens.

While some bacterial taxa tended to increase in thermally stressed colonies, such as *Pseudoalteromonas*, most of the major changes in the bacterial communities appeared colony-specific, explaining the great dispersion in beta diversity observed. As a result, we were unable to identify the microbe(s) responsible for the necrosis. This observation is in line with the Anna Karenina principle (59), which posits that the dysbiotic state of corals is the result of stochastic changes rather than unidirectional shifts, leading to more variation among stressed than healthy colonies. Directional shifts would rather be expected in the case of a role of the microbiota in the acclimation of the holobiont to changing conditions.

### Conclusion.

Gorgonians are of high ecological value as they create structural complexity in the benthic communities and thereby in the habitat of many species in the Mediterranean Sea. Elevated seawater temperatures may, however, threaten gorgonian populations and their ecological importance. In this study, we investigated the response of the holobiont of two Mediterranean gorgonian species during a thermal stress event, by following their physiology and the composition of their bacterial communities. Our results show a difference in thermotolerance between the gorgonians *P. clavata* and *E. cavolini*, which could be linked to their investment in antioxidant defense capacity and the differential impact on their energy reserves. This is consistent with field observations following mass mortality events. The gorgonian-associated bacterial community likely played only a minor role in holobiont acclimation to elevated temperatures in both species as only some minor shifts were observed in the initial weeks. This suggests that both *E. cavolini* and *P. clavata* are primarily “microbiome regulators,” trying to maintain a stable microbiota under stressful conditions. However, the significant depletion of energy stores and the inability to feed after prolonged thermal stress may have led to a reduced defense and microbiota regulatory capacity and ultimately major stochastic changes in the microbiota of *P. clavata* and partial colony mortality. Changes in the microbiota were primarily reflected by the significant reduction in the relative abundance of the coral’s main bacterial symbiont *Endozoicomonas*. Consequently, this likely impacted the beneficial functions in nutrition and defense that it provides to its host, which may have led to additional adverse impacts on holobiont metabolism and resilience, allowing opportunistic bacteria to proliferate. In contrast, the more tolerant *E. cavolini* continued to feed regardless of thermal stress, which likely contributed to a slower decrease in energy reserves during stress, and its higher antioxidant defense capability likely sufficiently protected against the adverse effects of thermal stress, allowing this gorgonian to maintain relative stability within the holobiont and prevent mortality. Although changes in the microbiota were observed, they were of a temporal nature, showing that the holobiont was capable of reverting potentially adverse shifts. Overall, our results suggest that gorgonian species are differentially impacted by thermal stress events, and that significant differences in stress resilience exist among individuals. Given their high ecological importance, higher than usual seawater temperatures due to climate change may thus induce major shifts in benthic community structures in the Mediterranean Sea.

## MATERIALS AND METHODS

### Biological material and summer thermal regime at collection site.

Nine colonies of both the gorgonian species *Eunicella cavolini* and *Paramuricea clavata* (15 cm long and 30 cm wide) were collected on SCUBA from populations at 35 m depth at Cap Caveau (43°15.615'N/5°17.357'E) and Tiboulen du Frioul (43°16.834'N/5°17.139'E; Marseille, France), respectively, on the 13th of July 2020. Fragments of gorgonian colonies were collected with the authorization of the Direction Interrégionale de la Mer Mediterranée (France).

The gorgonians were brought to the aquarium facility at the Centre Scientifique de Monaco, and maintained for 2 days in several large (100 L) open flow aquaria in the dark at 15–16°C, before being distributed over the experimental setup as described below.

The temperature of the seawater surrounding the gorgonians was 15°C at the time of collection, based on the dive computers of the divers. At this depth, the seawater temperature may increase to ∼24°C between mid-July and the end of August (Fig. S1). Temperatures ≥ 24°C are considered as thermal anomalies in the Mediterranean Sea. Besides, upwelling events of dense, cold water coming from the deep occur periodically because of the wind conditions at the site of collection. This may decrease seawater temperatures by up to 10°C within a few hours, and it can then slowly warm up again over a period of a week (7, 8, 60) (Fig. S1).

### Experimental design.

**FIG 5:**
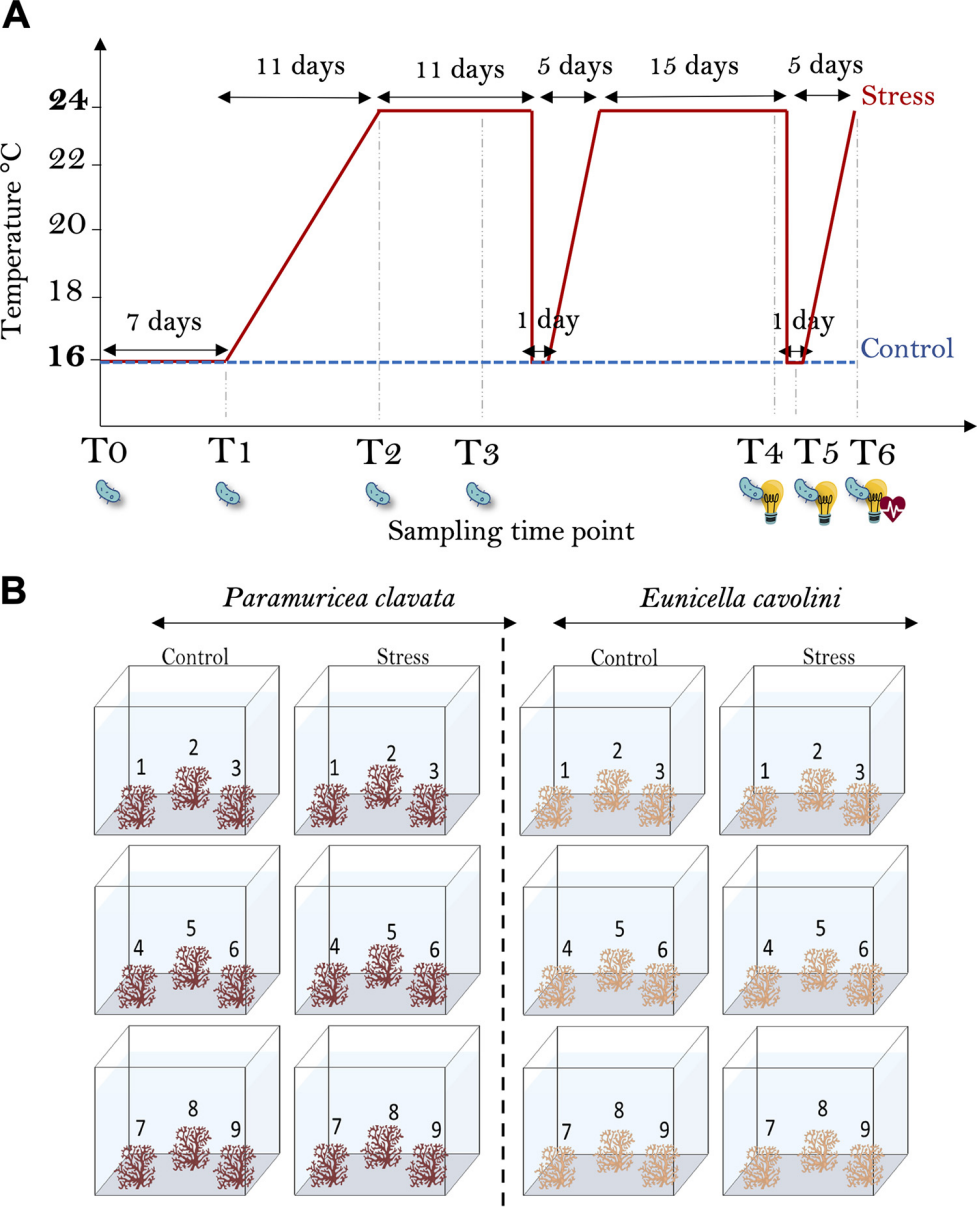
Experimental setup. Experimental temperature regime for Control (16°C) and Stress (24°C with upwelling) treatments applied to the gorgonians, with the sample collection time points (T0–T6) for physiological (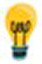 and 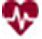 for total antioxidant capacity and coral energetics measurements, respectively) and molecular biology (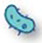 analyses indicated) (A). For each gorgonian species (*Paramuricea clavata* and *Eunicella cavolini*), nine colonies were cut into two equal-sized fragments. Paired fragments were divided over the two temperature conditions (“Control 16°C” and “Stress 24°C with upwelling”), with a maximum of three different colony fragments per tank (B).

To assess the impact of thermal stress on the gorgonian physiology and microbiota, gorgonians were exposed to two thermal conditions. Under control conditions, the temperature was kept at 15–16°C throughout the experiment, consistent with the temperature at the time of collection. Thermal stress conditions mimicked the *in situ* seawater temperature changes observed at the site of collection in summer: temperature was gradually increased from 16°C to 24°C in 11 days, maintained at 24°C during the subsequent 11 days, and was followed by two simulated upwelling events with a 15-day interval (Fig. 5A). A thermal stress temperature of 24°C was chosen as this has previously caused mortality in gorgonian populations *in situ* (7, 8) and experimental conditions (61) and is of ecological relevance for the populations at 35 m depth from which our corals were sourced.

The experimental setup consisted of 12 25-L aquaria, which were kept in the dark for the duration of the experiment. The aquaria received a continuous flow (15 L/h) of seawater pumped from 50-m depth, which was filtered through a 5 μm filter and a charcoal filter and treated with UV light to eliminate microbes. In each aquarium, a pump ensured continuous mixing of the seawater, and a heater, controlled by a temperature controller, maintained a constant seawater temperature. Both gorgonian species were fed with cyclops, red plankton, and artemia, and the aquaria were cleaned once a week.

Six aquaria were assigned to each gorgonian species, with three aquaria for each thermal condition. The two gorgonian species were kept in separate tanks to avoid interspecies interactions and competition (62). Each colony was cut into two equal-sized fragments, and the fragments were divided over the two temperature conditions, ensuring that for each thermally stressed coral a paired control sample was present, allowing statistical corrections for colony (Fig. 5B). The fragments were then distributed over the aquaria, ensuring one fragment per colony per thermal condition and three fragments per aquarium (Fig. 5B). The gorgonians were kept at 16°C for 1 week to acclimate prior to the start of the experiment (Fig. 5A). Samples from each fragment were taken at seven time points over the course of the experiment (Fig. 5A) for physiology and molecular biology analyses as detailed below.

### Host physiological parameters. (i) Coral energetics.

**(a) Sample preparation.** Samples of around 6 cm were collected at the end of the experiment (T6 in Fig. 5A) from each fragment and immediately flash frozen and stored at −80°C until further processing. Frozen samples were lyophilized overnight (Christ Martin Alpha Freeze dryer, Fisher Scientific). The tissue was removed from the skeleton and crushed into a powder. The total dry weight (DW) of the gorgonian tissue (i.e., excluding the proteinaceous skeleton) was determined. Subsamples with a known weight were then used to perform the different analyses described below.

Ash free dry weight (AFDW) was used to normalize the physiological parameters and for interspecies comparisons (63). For this purpose, a correlation curve between DW and AFDW was established for each gorgonian species. Subsamples with different DW (from 2 to 15 mg) were weighed before and after being burned at 450°C for 4 h to remove all the organic material. The difference between the two measurements (total dry weight and ash) corresponds to the AFDW.

**(b) Total protein, lipid, carbohydrate, and lactate concentration.** Proteins were extracted from 5 mg of dry tissue in 200 μL of 1 M sodium hydroxide and heated at 90°C for 30 min. Total protein concentration was measured using the Pierce BCA Protein assay kit (Thermo Fisher Scientific) following the manufacturer’s instructions and expressed in mg of proteins per mg AFDW.

To extract lipids, 10 mg of dry tissue was added to 1.5 mL of chloroform/methanol (2v:1v) in Pyrex tubes and incubated at room temperature for 20 min on an orbital shaker. The tubes were centrifuged at 1,000 g/min for 5 min to remove debris, and 1 mL of extract was subsequently evaporated in a dry bath at 90°C. Total lipid concentration was measured using a protocol based on the sulfo-phospho-vanillin reaction method from Barnes and Blackstock (64). The net absorbance values at 520 nm were read using a spectrofluorometer (Xenius, SAFAS, Monaco) and were compared with a standard curve made of known concentrations of cholesterol (65). Total lipid concentrations were expressed in mg of lipids per mg AFDW.

Total carbohydrates were extracted from 15 mg of dry tissue by sonication (20 pulses) in 200 μL of cold 1× PBS on ice. Total carbohydrate concentration was measured using the Total Carbohydrate assay kit (Sigma-Aldrich). Kit standards were diluted in cold 1× PBS. The net absorbance values at 490 nm were read using a spectrofluorometer (Xenius, SAFAS, Monaco) and were compared with a standard curve made of known concentrations of d-glucose. Total carbohydrate concentrations were expressed in μg of carbohydrates per mg AFDW.

Lactate concentration was measured in 10 mg of dry tissue using the Lactate assay kit (SigmaAldrich), which uses lactate dehydrogenase enzyme activity to convert lactate into pyruvate followed by the colorimetric detection (net absorbance values read at 570 nm). Lactate concentration was expressed in ng per mg AFDW.

### (ii) Total antioxidant capacity (TAC).

Samples of 2 cm were collected from each colony at T4, T5, and T6 (i.e., before, during, and after the second simulated upwelling event; Fig. 5A). Samples were flash frozen and stored at −80°C. Frozen samples were put in a 1.5 mL Eppendorf tube containing 200 μL of cold 1× PBS (Phosphate-Buffered Saline), and tissues were lysed by sonication (20 pulses at 70 Hz; Vibra-Cell, Bioblock Scientific, France) on ice. To normalize TAC values, the protein concentration in 5 μL of sample was also assessed using the Bradford Protein assay kit (Thermo Fisher). The lysate was diluted to the same protein concentration, and the total antioxidant capacity (or oxyradical scavenging capacity) was determined using the OxiSelect Total Antioxidant Capacity assay kit (Cell Biolabs Inc.). The net absorbance values at 490 nm were read using a spectrofluorometer (Xenius, SAFAS, Monaco) and were compared with a standard curve made of known concentrations of the antioxidant uric acid. The results are expressed as μM Copper Reducing Equivalents (CRE) normalized per mg of protein.

### (iii) Data analyses.

All graphical and statistical analyses were carried out in the R environment (version 4.0.0). Generalized linear mixed effects models (R-package *lme4*) (66) were fitted including *treatment* and/or *time* as fixed factors and *experimental tank* and *colony* as random factors to assess whether an effect of tank and/or colony existed in the measured physiological parameters (protein content, lipid content, carbohydrate content, TAC concentration). As no significant random effects were observed, all data were analyzed using generalized linear models (GLM) with *treatment* and/or *time* as fixed factors. Normality and homoscedasticity of the residuals were verified using the Shapiro’s and Levene’s tests (R-package *car*) (67), respectively. In case the distribution of residuals did not follow a normal distribution, a Box Cox-based transformation was applied prior to model fitting. In case of multiple hypothesis testing, a Bonferroni correction was applied and an α of < 0.025 was used.

### Bacterial community analysis. (i) DNA extraction.

Samples of 2 cm were collected from each colony at all time points (Fig. 5A) and kept in RNA later at 4°C until further processing. DNA was extracted using the DNeasy PowerBiofilm kit (Qiagen) with the following modifications: during the cell lysis step, 2 μL of Proteinase K (600 U/mL) was added to the sample and incubated at 60°C for 2 h, followed by 2 min of bead beating using the CryoMill (Retch, Germany) at a frequency of 30 Hz. Negative extraction control samples (i.e., extraction without sample material) were processed at the same time as the samples of gorgonians to account for contaminants. DNA concentration was measured using a fluorimeter (BioTek), and DNA was stored at −20°C.

### (ii) Illumina MiSeq 16S rRNA gene amplicon sequencing.

DNA was sent to STAB VIDA (Portugal) for amplicon library preparation using Illumina’s standard 16S Metagenomic Sequencing Library Preparation protocol (68). The V3-V4 region of the *16S rRNA* gene was amplified using the forward primer 341F 5′-CCTACGGGNGGCWGCAG-3′ and the reverse primer 785R 5′-GACTACHVGGGTATCTAATCC-3′ (69). Libraries were pooled in equimolar ratios and paired-end (2 × 300 bp) sequenced on the Illumina MiSeq platform with V3 chemistry. The .fastq files containing the raw sequencing data have been deposited in the NCBI’s Short Read Archive (SRA) under BioProject accession numbers PRJNA746505 and PRJNA748844.

### (iii) Bioinformatics data processing.

The 16S rRNA gene amplicon data were processed using the USEARCH v11 software (64-bit version, https://drive5.com/usearch/) (70). MiSeq sequencing produced 24,054,550 reads, ranging from 20,762 to 169,948 reads per sample, and 4792 and 4938 reads in the negative extraction control samples. Reverse reads were truncated using -fastx_truncate with a base quality threshold of >Q20 to ensure that high quality reverse read (R2) data were used for merging. Forward read (R1) and reverse reads (R2) were merged using -fastq_mergepairs with the following settings: a minimum and maximum length of the merged sequence of 410 bp and 500 bp, and a minimum and maximum overlap length of 20 bp and 100 bp. The primer sequences were removed from the merged sequences using -fastq_truncate, and the resulting sequences were quality filtered allowing a maximum of one expected error using -fastq_filter, obtaining 9,163,472 sequences of 427 bp on average. A total of 500,997 unique sequences were identified and subsequently denoised using the UNOISE3 algorithm, obtaining 2512 zero-radius operational taxonomic units (zOTUs). zOTUs were annotated using the SINTAX algorithm with an assignment confidence cutoff of 0.6 based on the SILVA SSU reference database (version 138) (71). OTU tables were generated by clustering sequences with an identity similarity value of 0.985 using -usearch_global algorithm. The OTU table, the sequences of each zOTU, and the metadata are available as supplemental data.

### (iv) Data analyses.

The R-package *decontam* (72) was used to identify putative contaminant OTUs in the samples based on the negative extraction control samples (*isContaminant* function). Nineteen of the 2,196 OTUs were identified as putative contaminants and were removed. Chao1 estimations of the richness were calculated using the R-package *vegan* (73), and analyses of variance (ANOVA) were used to test if Chao1 estimation richness was similar between thermal conditions and gorgonian species (Table S1). For beta diversity analyses, raw OTU abundances were transformed by calculating the centered log-ratios (clr) as implemented in the R-package *compositions* (74) after zero counts were imputed based on Bayesian multiplicative replacement (Bayes-LaPlace BM method of the *cmultRepl* function of the R-package *zCompositions*) (75). Clr-transformed count data were then used as inputs for multivariate hypothesis testing (76, 77). First, an Aitchison distance matrix was generated by calculating the Euclidean distances between samples based on the clr-transformed data table. Based on the Aitchison distance matrix, principal-component analyses (PCA), as well as permutational multivariate analysis of variance (PERMANOVA) and dispersion analyses (PERMDISP) as implemented in PRIMER6 + PERMANOVA (78, 79), were used to assess differences in beta diversity and dispersion between the two thermal conditions and sampling time points. The PERMANOVA model included *Thermal Condition* and *Time* (nested in *Thermal Condition*) as fixed factors and *Colony* as a random factor to account for repeated measures, and the analysis was performed under Type III partial sums of squares under a reduced model with 9,999 permutations. In addition, as changes in the bacterial communities appeared to be colony dependent, hierarchical cluster analyses (WardD2 method) were used to objectively identify colonies that showed large or minor changes in the microbiota and assign those to two different groups (A and B). Taking into account the two groups, PERMANOVA and dispersion analyses were performed to test for differences in beta diversity of the microbiota due to treatment at different sampling time points (T4–T6) and for temporal effects within thermal treatments.

Distance-based redundancy analyses (dbRDA) were also carried out to evaluate whether links existed between coral host physiological parameters (i.e., protein, lipid, carbohydrate concentration, and TAC) as well as biomass loss (i.e., percentage of protein, lipid, and carbohydrate lost between control and stress colonies), and the differences in the bacterial community structure at time point T6.

Differential abundance analyses were performed to identify OTUs that were differentially abundant between treatments in both groups (i.e., A and B groups) at time points T4, T5, and T6 using the R-package *ANCOMBC*, excluding OTUs with a proportion of zeroes greater than 0.6 and considering OTUs differentially abundant when α < 0.01 (80).

## References

[1] Giorgi F. 2006. Climate change hot‐spots. Geophys Res Lett 33:L08707. 10.1029/2006GL025734.

[2] Garrabou J, Ledoux J-B, Bensoussan N, Gómez-Gras D, Linares C. 2021. Sliding toward the collapse of Mediterranean coastal marine rocky ecosystems, p 291–324. *In* Canadell JG, Jackson RB (ed), Ecosystem collapse and climate change. Springer International Publishing, Cham, Switzerland. 10.1007/978-3-030-71330-0_11.

[3] Cherif S, Doblas-Miranda E, Lionello P, Borrego C, Giorgi F, Iglesias A, Jebari S, Mahmoudi E, Moriondo M, Pringault O, Rilov G, Somot S, Tsikliras A, Vilà M, Zittis G. 2021. Drivers of change. *In* Cramer W, Guiot J, Marini K (ed), Climate and environmental change in the Mediterranean basin—current situation and risks for the future. Union for the Mediterranean, Marseille, France.

[4] Ponti M, Turicchia E, Costantini F, Gori A, Bramanti L, Di Camillo CG, Linares C, Rossi S, Abbiati M, Garrabou J, Cerrano C. 2019. Mediterranean gorgonian forests: distribution patterns and ecological roles. Abstr 3ème Symposium Méditerranéen sur la conservation du Coralligène at autres Bio-Concrétions, Antalya, Turquie, 15–16 January 2019.

[5] Ponti M, Perlini RA, Ventra V, Grech D, Abbiati M, Cerrano C. 2014. Ecological shifts in Mediterranean coralligenous assemblages related to gorgonian forest loss. PLoS One 9:e102782. 10.1371/journal.pone.0102782. 25054286 PMC4108394

[6] Garrabou J, Gómez-Gras D, Ledoux J-B, Linares C, Bensoussan N, López-Sendino P, Bazairi H, Espinosa F, Ramdani M, Grimes S, Benabdi M, Souissi JB, Soufi E, Khamassi F, Ghanem R, Ocaña O, Ramos-Esplà A, Izquierdo A, Anton I, Rubio-Portillo E, Barbera C, Cebrian E, Marbà N, Hendriks IE, Duarte CM, Deudero S, Díaz D, Vázquez-Luis M, Alvarez E, Hereu B, Kersting DK, Gori A, Viladrich N, Sartoretto S, Pairaud I, Ruitton S, Pergent G, Pergent-Martini C, Rouanet E, Teixidó N, Gattuso J-P, Fraschetti S, Rivetti I, Azzurro E, Cerrano C, Ponti M, Turicchia E, Bavestrello G, Cattaneo-Vietti R, Bo M, et al. 2019. collaborative database to track mass mortality events in the Mediterranean Sea. Front Mar Sci 6. 10.3389/fmars.2019.00707.

[7] Garrabou J, Coma R, Bensoussan N, Bally M, Chevaldonné P, Cigliano M, Diaz D, Harmelin JG, Gambi MC, Kersting DK, Ledoux JB, Lejeusne C, Linares C, Marschal C, Pérez T, Ribes M, Romano JC, Serrano E, Teixido N, Torrents O, Zabala M, Zuberer F, Cerrano C. 2009. Mass mortality in Northwestern Mediterranean rocky benthic communities: effects of the 2003 heat wave. Global Change Biology 15:1090–1103. 10.1111/j.1365-2486.2008.01823.x.

[8] Crisci C, Bensoussan N, Romano J-C, Garrabou J. 2011. Temperature anomalies and mortality events in marine communities: insights on factors behind differential mortality impacts in the NW Mediterranean. PLoS One 6:e23814. 10.1371/journal.pone.0023814. 21931615 PMC3171413

[9] Pairaud IL, Gatti J, Bensoussan N, Verney R, Garreau P. 2011. Hydrology and circulation in a coastal area off Marseille: validation of a nested 3D model with observations. J Marine Systems 88:20–33. 10.1016/j.jmarsys.2011.02.010.

[10] Cau A, Bramanti L, Cannas R, Moccia D, Padedda B, Porcu C, Sacco F, Follesa M. 2018. Differential response to thermal stress of shallow and deep dwelling colonies of Mediterranean red coral *Corallium rubrum* (L., 1758). Adv Ocean Limnol 9. 10.4081/aiol.2018.7275.

[11] Kipson S, Linares C, Teixidó N, Bakran-Petricioli T, Garrabou J. 2012. Effects of thermal stress on early development stages of a gorgonian coral. Mar Ecol Prog Ser 470:69–78. 10.3354/meps09982.

[12] Ezzat L, Merle P-L, Furla P, Buttler A, Ferrier-Pagès C. 2013. The response of the Mediterranean gorgonian *Eunicella singularis* to thermal stress is independent of its nutritional regime. PLoS One 8:e64370. 10.1371/journal.pone.0064370. 23667711 PMC3648542

[13] Ferrier-Pagès C, Tambutté E, Zamoum T, Segonds N, Merle PL, Bensoussan N, Allemand D, Garrabou J, Tambutté S. 2009. Physiological response of the symbiotic gorgonian *Eunicella singularis* to a long-term temperature increase. J Exp Biol 212:3007–3015. 10.1242/jeb.031823. 19717684

[14] Previati M, Scinto A, Cerrano C, Osinga R. 2010. Oxygen consumption in Mediterranean octocorals under different temperatures. J Experimental Marine Biology and Ecology 390:39–48. 10.1016/j.jembe.2010.04.025.

[15] Linares C, Cebrian E, Kipson S, Garrabou J. 2013. Does thermal history influence the tolerance of temperate gorgonians to future warming? Mar Environ Res 89:45–52. 10.1016/j.marenvres.2013.04.009. 23735816

[16] Torrents O, Tambutté E, Caminiti N, Garrabou J. 2008. Upper thermal thresholds of shallow vs. deep populations of the precious Mediterranean red coral *Corallium rubrum* (L.): assessing the potential effects of warming in the NW Mediterranean. J Experimental Marine Biology and Ecology 357:7–19. 10.1016/j.jembe.2007.12.006.

[17] Pivotto ID, Nerini D, Masmoudi M, Kara H, Chaoui L, Aurelle D. 2015. Highly contrasted responses of Mediterranean octocorals to climate change along a depth gradient. R Soc Open Sci 2:140493. 10.1098/rsos.140493. 26064654 PMC4453260

[18] Pratlong M, Haguenauer A, Chabrol O, Klopp C, Pontarotti P, Aurelle D. 2015. The red coral (*Corallium rubrum*) transcriptome: a new resource for population genetics and local adaptation studies. Mol Ecol Resour 15:1205–1215. 10.1111/1755-0998.12383. 25648864

[19] Ledoux J-B, Aurelle D, Bensoussan N, Marschal C, Féral J-P, Garrabou J. 2015. Potential for adaptive evolution at species range margins: contrasting interactions between red coral populations and their environment in a changing ocean. Ecol Evol 5:1178–1192. 10.1002/ece3.1324. 25859324 PMC4377262

[20] Crisci C, Ledoux JB, Mokhtar- Jamaï K, Bally M, Bensoussan N, Aurelle D, Cebrian E, Coma R, Féral JP, La Rivière M, Linares C, López-Sendino P, Marschal C, Ribes M, Teixidó N, Zuberer F, Garrabou J. 2017. Regional and local environmental conditions do not shape the response to warming of a marine habitat-forming species. Sci Rep 7:5069. 10.1038/s41598-017-05220-4. 28698582 PMC5505982

[21] Marangoni L, Rottier C, Ferrier-Pagès C. 2021. Symbiont regulation in *Stylophora pistillata* during cold stress: an acclimation mechanism against oxidative stress and severe bleaching. The J Experimental Biology 224:jeb.235275. 10.1242/jeb.235275. 33431596

[22] Marangoni LFdB, Dalmolin C, Marques JA, Klein RD, Abrantes DP, Pereira CM, Calderon EN, Castro CBe, Bianchini A. 2019. Oxidative stress biomarkers as potential tools in reef degradation monitoring: a study case in a South Atlantic reef under influence of the 2015–2016 El Niño/Southern Oscillation (ENSO). Ecological Indicators 106:105533. 10.1016/j.ecolind.2019.105533.

[23] Gardner SG, Raina J-B, Nitschke MR, Nielsen DA, Stat M, Motti CA, Ralph PJ, Petrou K. 2017. A multi-trait systems approach reveals a response cascade to bleaching in corals. BMC Biol 15:117. 10.1186/s12915-017-0459-2. 29216891 PMC5719617

[24] Gardner SG, Nielsen DA, Laczka O, Shimmon R, Beltran VH, Ralph PJ, Petrou K. 2016. Dimethylsulfoniopropionate, superoxide dismutase and glutathione as stress response indicators in three corals under short-term hyposalinity stress. Proc R Soc B 283:20152418. 10.1098/rspb.2015.2418. PMC4760162 26865302

[25] Richier S, Cottalorda J-M, Guillaume MMM, Fernandez C, Allemand D, Furla P. 2008. Depth-dependant response to light of the reef building coral, *Pocillopora verrucosa*: implication of oxidative stress. J Experimental Marine Biology and Ecology 357:48–56. 10.1016/j.jembe.2007.12.026.

[26] Lesser MP. 2006. Oxidative stress in marine environments: biochemistry and physiological ecology. Annu Rev Physiol 68:253–278. 10.1146/annurev.physiol.68.040104.110001. 16460273

[27] Lesser MP. 2011. Coral bleaching: causes and mechanisms, p 405–419. *In* Dubinsky Z, Stambler N (ed), Coral reefs: an ecosystem in transition. Springer, Dordrecht, the Netherlands. 10.1007/978-94-007-0114-4_23.

[28] Knowlton N, Rohwer F. 2003. Multispecies microbial mutualisms on coral reefs: the host as a habitat. Am Nat 162:S51–S62. 10.1086/378684. 14583857

[29] van de Water JA, Melkonian R, Junca H, Voolstra CR, Reynaud S, Allemand D, Ferrier-Pagès C. 2016. Spirochaetes dominate the microbial community associated with the red coral *Corallium rubrum* on a broad geographic scale. Sci Rep 6:27277. 10.1038/srep27277. 27263657 PMC4893704

[30] van de Water JAJM, Allemand D, Ferrier-Pagès C. 2018. Host-microbe interactions in octocoral holobionts—recent advances and perspectives. Microbiome 6:64. 10.1186/s40168-018-0431-6. 29609655 PMC5880021

[31] van de Water JAJM, Melkonian R, Voolstra CR, Junca H, Beraud E, Allemand D, Ferrier-Pagès C. 2017. Comparative assessment of Mediterranean gorgonian-associated microbial communities reveals conserved core and locally variant bacteria. Microb Ecol 73:466–478. 10.1007/s00248-016-0858-x. 27726033

[32] Neave MJ, Apprill A, Ferrier-Pagès C, Voolstra CR. 2016. Diversity and function of prevalent symbiotic marine bacteria in the genus *Endozoicomonas*. Appl Microbiol Biotechnol 100:8315–8324. 10.1007/s00253-016-7777-0. 27557714 PMC5018254

[33] Voolstra CR, Ziegler M. 2020. Adapting with microbial help: microbiome flexibility facilitates rapid responses to environmental change. Bioessays 42:e2000004. 10.1002/bies.202000004. 32548850

[34] Damjanovic K, Blackall LL, Webster NS, van Oppen MJH. 2017. The contribution of microbial biotechnology to mitigating coral reef degradation. Microb Biotechnol 10:1236–1243. 10.1111/1751-7915.12769. 28696067 PMC5609283

[35] Peixoto RS, Rosado PM, Leite DCdA, Rosado AS, Bourne DG. 2017. Beneficial Microorganisms for Corals (BMC): proposed mechanisms for coral health and resilience. Front Microbiol 8:341. 10.3389/fmicb.2017.00341. 28326066 PMC5339234

[36] McDevitt-Irwin JM, Baum JK, Garren M, Vega Thurber RL. 2017. Responses of coral-associated bacterial communities to local and global stressors. Front Mar Sci 4. 10.3389/fmars.2017.00262.

[37] Ziegler M, Seneca FO, Yum LK, Palumbi SR, Voolstra CR. 2017. Bacterial community dynamics are linked to patterns of coral heat tolerance. Nat Commun 8:14213. 10.1038/ncomms14213. 28186132 PMC5309854

[38] Röthig T, Ochsenkühn MA, Roik A, van der Merwe R, Voolstra CR. 2016. Long-term salinity tolerance is accompanied by major restructuring of the coral bacterial microbiome. Mol Ecol 25:1308–1323. 10.1111/mec.13567. 26840035 PMC4804745

[39] Pogoreutz C, Rädecker N, Cárdenas A, Gärdes A, Wild C, Voolstra CR. 2018. Dominance of *Endozoicomonas* bacteria throughout coral bleaching and mortality suggests structural inflexibility of the *Pocillopora verrucosa* microbiome. Ecol Evol 8:2240–2252. 10.1002/ece3.3830. 29468040 PMC5817147

[40] Gajigan AP, Diaz LA, Conaco C. 2017. Resilience of the prokaryotic microbial community of *Acropora digitifera* to elevated temperature. Microbiologyopen 6:e00478. 10.1002/mbo3.478. PMC5552946 28425179

[41] Lee STM, Davy SK, Tang S-L, Kench PS. 2017. Water flow buffers shifts in bacterial community structure in heat-stressed *Acropora muricata*. Sci Rep 7:43600. 10.1038/srep43600. 28240318 PMC5327421

[42] Grottoli AG, Dalcin Martins P, Wilkins MJ, Johnston MD, Warner ME, Cai W-J, Melman TF, Hoadley KD, Pettay DT, Levas S, Schoepf V. 2018. Coral physiology and microbiome dynamics under combined warming and ocean acidification. PLoS One 13:e0191156. 10.1371/journal.pone.0191156. 29338021 PMC5770069

[43] Tracy AM, Koren O, Douglas N, Weil E, Harvell CD. 2015. Persistent shifts in Caribbean coral microbiota are linked to the 2010 warm thermal anomaly. Environ Microbiol Rep 7:471–479. 10.1111/1758-2229.12274. 25683053

[44] Epstein HE, Smith HA, Torda G, Oppen MJ. 2019. Microbiome engineering: enhancing climate resilience in corals. Front Ecol Environ 17:100–108. 10.1002/fee.2001.

[45] Liñán-Cabello MA, Flores-Ramírez LA, Zenteno-Savin T, Olguín-Monroy NO, Sosa-Avalos R, Patiño-Barragan M, Olivos-Ortiz A. 2009. Seasonal changes of antioxidant and oxidative parameters in the coral *Pocillopora capitata* on the Pacific coast of Mexico. Marine Ecology 31:407–417. 10.1111/j.1439-0485.2009.00349.x.

[46] Haguenauer A, Zuberer F, Ledoux J-B, Aurelle D. 2013. Adaptive abilities of the Mediterranean red coral *Corallium rubrum* in a heterogeneous and changing environment: from population to functional genetics. J Experimental Marine Biology and Ecology 449:349–357. 10.1016/j.jembe.2013.10.010.

[47] Sini M, Kipson S, Linares C, Koutsoubas D, Garrabou J. 2015. The yellow gorgonian *Eunicella cavolini*: demography and disturbance levels across the Mediterranean Sea. PLoS One 10:e0126253. 10.1371/journal.pone.0126253. 25942319 PMC4420262

[48] Rubio-Portillo E, Ramos-Esplá AA, Antón J. 2021. Shifts in marine invertebrate bacterial assemblages associated with tissue necrosis during a heat wave. Coral Reefs 40:395–404. 10.1007/s00338-021-02075-0.

[49] Coma R, Ribes M, Serrano E, Jiménez E, Salat J, Pascual J. 2009. Global warming-enhanced stratification and mass mortality events in the Mediterranean. Proc Natl Acad Sci USA 106:6176–6181. 10.1073/pnas.0805801106. 19332777 PMC2669359

[50] Jessen C, Villa Lizcano JF, Bayer T, Roder C, Aranda M, Wild C, Voolstra CR. 2013. *In-situ* effects of eutrophication and overfishing on physiology and bacterial diversity of the Red Sea coral *Acropora hemprichii*. PLoS One 8:e62091. 10.1371/journal.pone.0062091. 23630625 PMC3632597

[51] Ziegler M, Grupstra CGB, Barreto MM, Eaton M, BaOmar J, Zubier K, Al-Sofyani A, Turki AJ, Ormond R, Voolstra CR. 2019. Coral bacterial community structure responds to environmental change in a host-specific manner. Nat Commun 10:3092. 10.1038/s41467-019-10969-5. 31300639 PMC6626051

[52] van de Water JAJM, Coppari M, Enrichetti F, Ferrier-Pagès C, Bo M. 2020. Local conditions influence the prokaryotic communities associated with the mesophotic black coral *Antipathella subpinnata*. Front Microbiol 11. 10.3389/fmicb.2020.537813. PMC7573217 33123099

[53] Keller-Costa T, Lago-Lestón A, Saraiva JP, Toscan R, Silva SG, Gonçalves J, Cox CJ, Kyrpides N, Nunes da Rocha U, Costa R. 2021. Metagenomic insights into the taxonomy, function, and dysbiosis of prokaryotic communities in octocorals. Microbiome 9:72. 10.1186/s40168-021-01031-y. 33766108 PMC7993494

[54] Apprill A, Hughen K, Mincer T. 2013. Major similarities in the bacterial communities associated with lesioned and healthy Fungiidae corals. Environ Microbiol 15:2063–2072. 10.1111/1462-2920.12107. 23516962

[55] Beurmann S, Ushijima B, Videau P, Svoboda CM, Smith AM, Rivers OS, Aeby GS, Callahan SM. 2017. *Pseudoalteromonas piratica* strain OCN003 is a coral pathogen that causes a switch from chronic to acute *Montipora* white syndrome in *Montipora capitata*. PLoS One 12:e0188319. 10.1371/journal.pone.0188319. 29145488 PMC5690655

[56] Munn CB. 2015. The role of Vibrios in diseases of corals. Microbiol Spectr 3. 10.1128/microbiolspec.VE-0006-2014. 26350314

[57] Miura N, Motone K, Takagi T, Aburaya S, Watanabe S, Aoki W, Ueda M. 2019. *Ruegeria* sp. strains isolated from the reef-building coral *Galaxea fascicularis* inhibit growth of the temperature-dependent pathogen *Vibrio coralliilyticus*. Mar Biotechnol (NY) 21:1–8. 10.1007/s10126-018-9853-1. 30194504

[58] Rosado PM, Leite DCA, Duarte GAS, Chaloub RM, Jospin G, Nunes da Rocha U, Saraiva J, Dini-Andreote F, Eisen JA, Bourne DG, Peixoto RS. 2019. Marine probiotics: increasing coral resistance to bleaching through microbiome manipulation. ISME J 13:921–936. 10.1038/s41396-018-0323-6. 30518818 PMC6461899

[59] Zaneveld JR, McMinds R, Vega Thurber R. 2017. Stress and stability: applying the Anna Karenina principle to animal microbiomes. Nat Microbiol 2:17121. 10.1038/nmicrobiol.2017.121. 28836573

[60] Bensoussan N, Romano J-C, Harmelin J-G, Garrabou J. 2010. High resolution characterization of northwest Mediterranean coastal waters thermal regimes: to better understand responses of benthic communities to climate change. Estuarine, Coastal and Shelf Science 87:431–441. 10.1016/j.ecss.2010.01.008.

[61] Bally M, Garrabou J. 2007. Thermodependent bacterial pathogens and mass mortalities in temperate benthic communities: a new case of emerging disease linked to climate change. Global Change Biol 13:2078–2088. 10.1111/j.1365-2486.2007.01423.x.

[62] Turicchia E, Abbiati M, Ponti M. 2020. Mediterranean gorgonians fighting. Mar Biodivers 50:33. 10.1007/s12526-020-01064-w.

[63] Pupier CA, Fine M, Bednarz VN, Rottier C, Grover R, Ferrier-Pagès C. 2019. Productivity and carbon fluxes depend on species and symbiont density in soft coral symbioses. Sci Rep 9:17819. 10.1038/s41598-019-54209-8. 31780787 PMC6882883

[64] Barnes H, Blackstock J. 1973. Estimation of lipids in marine animals and tissues: detailed investigation of the sulphophosphovanilun method for “total” lipids. J Experimental Marine Biology and Ecology 12:103–118. 10.1016/0022-0981(73)90040-3.

[65] Knight JA, Anderson S, Rawle JM. 1972. Chemical basis of the sulfo-phospho-vanillin reaction for estimating total serum lipids. Clin Chem 18:199–202. 10.1093/clinchem/18.3.199. 5020813

[66] Bates D, Machler M, Bolker B, Walker S. 2015. Fitting linear mixed-effects models using lme4. J Stat Soft 67:1–48. 10.18637/jss.v067.i01.

[67] Fox J, Weisberg S. 2018. An R companion to applied regression. Sage, Thousand Oaks, CA.

[68] Illumina. 2013. 16S Metagenomic sequencing library preparation. https://support.illumina.com/documents/documentation/chemistry_documentation/16s/16s-metagenomic-library-prep-guide-15044223-b.pdf.

[69] Klindworth A, Pruesse E, Schweer T, Peplies J, Quast C, Horn M, Glöckner FO. 2013. Evaluation of general 16S ribosomal RNA gene PCR primers for classical and next-generation sequencing-based diversity studies. Nucleic Acids Res 41:e1. 10.1093/nar/gks808. 22933715 PMC3592464

[70] Edgar RC. 2018. Taxonomy annotation and guide tree errors in 16S rRNA databases. bioRxiv 10.1101/288654. PMC6003391 29910992

[71] Quast C, Pruesse E, Yilmaz P, Gerken J, Schweer T, Yarza P, Peplies J, Glöckner FO. 2013. The SILVA ribosomal RNA gene database project: improved data processing and web-based tools. Nucleic Acids Res 41:D590–D596. 10.1093/nar/gks1219. 23193283 PMC3531112

[72] Davis NM, Proctor DM, Holmes SP, Relman DA, Callahan BJ. 2018. Simple statistical identification and removal of contaminant sequences in marker-gene and metagenomics data. bioRxiv 10.1101/221499. PMC6298009 30558668

[73] Oksanen J, Blanchet FG, Kindt R, Legendre P, Minchin P, O'Hara B, Simpson G, Solymos P, Stevens H, Wagner H. 2015. Vegan: community ecology package. R Package Version 22–1 2:1–2.

[74] van den Boogaart KG, Tolosana-Delgado R. 2008. “compositions”: a unified R package to analyze compositional data. Computers & Geosciences 34:320–338. 10.1016/j.cageo.2006.11.017.

[75] Palarea-Albaladejo J, Martín-Fernández JA. 2015. zCompositions—R package for multivariate imputation of left-censored data under a compositional approach. Chemometrics and Intelligent Laboratory Systems 143:85–96. 10.1016/j.chemolab.2015.02.019.

[76] Gloor GB, Macklaim JM, Pawlowsky-Glahn V, Egozcue JJ. 2017. Microbiome datasets are compositional: and this is not optional. Front Microbiol 8:2224–2224. 10.3389/fmicb.2017.02224. 29187837 PMC5695134

[77] Quinn TP, Erb I, Richardson MF, Crowley TM. 2018. Understanding sequencing data as compositions: an outlook and review. Bioinformatics 34:2870–2878. 10.1093/bioinformatics/bty175. 29608657 PMC6084572

[78] Anderson MJ, Walsh DCI. 2013. PERMANOVA, ANOSIM, and the Mantel test in the face of heterogeneous dispersions: what null hypothesis are you testing? Ecological Monographs 83:557–574. 10.1890/12-2010.1.

[79] Clarke K, Gorley RN. 2006. Primer v6: user manual/tutorial. PRIMER-E, Auckland, New Zealand.

[80] Lin H, Peddada SD. 2020. Analysis of compositions of microbiomes with bias correction. Nat Commun 11:3514. 10.1038/s41467-020-17041-7. 32665548 PMC7360769

