# Supplementary material for: The Effect of Thermal Stress on the Physiology and Bacterial Communities of Two Key Mediterranean Gorgonians
Source: Appl Environ Microbiol. 2022 Mar 22;88(6):e02340-21. doi: 10.1128/aem.02340-21 (PMC8939326; doi:10.1128/aem.02340-21)
Supplement: Supplemental file 1 — Fig. S1 to S8. Download aem.02340-21-s0001.pdf, PDF file, 3.9 MB [file aem.02340-21-s0001.pdf]

SUPPLEMENTARY INFORMATION

for

The effect of thermal stress on the physiology and bacterial communities of two key Mediterranean gorgonians

Romie Tignat-Perrier[1,2†], Jeroen A.J.M. van de Water[1,2†], Dorian Guillemain[4], Didier Aurelle[5,6], Denis Allemand[3], Christine Ferrier-Pagès[2]

Affiliations:

[1]Unité de Recherche sur la Biologie des Coraux Précieux CSM - CHANEL, Centre Scientifique de Monaco, 8 Quai Antoine 1er, MC 98000, Monaco, Principality of Monaco

[2]Coral EcophysiologyLaboratory, Centre Scientifique de Monaco, 8 Quai Antoine 1er, MC 98000, Monaco, Principality of Monaco

[3]Centre Scientifique de Monaco, 8 Quai Antoine 1er, MC 98000, Monaco, Principality of Monaco

[4]Aix Marseille Université, CNRS, IRD, IRSTEA, OSU Institut Pythéas, Marseille, France

[5]Aix Marseille Université, Université de Toulon, CNRS, IRD, MIO, Marseille, France

[6]Institut de Systématique, Evolution, Biodiversité (ISYEB), Muséum national d'Histoire naturelle, CNRS, Sorbonne Université, EPHE, Université des Antilles, 57 rue Cuvier, 75005 Paris, France

†These authors contributed equally to this work

Corresponding author: Romie Tignat-Perrier, romie.tignat@centrescientifique.mc

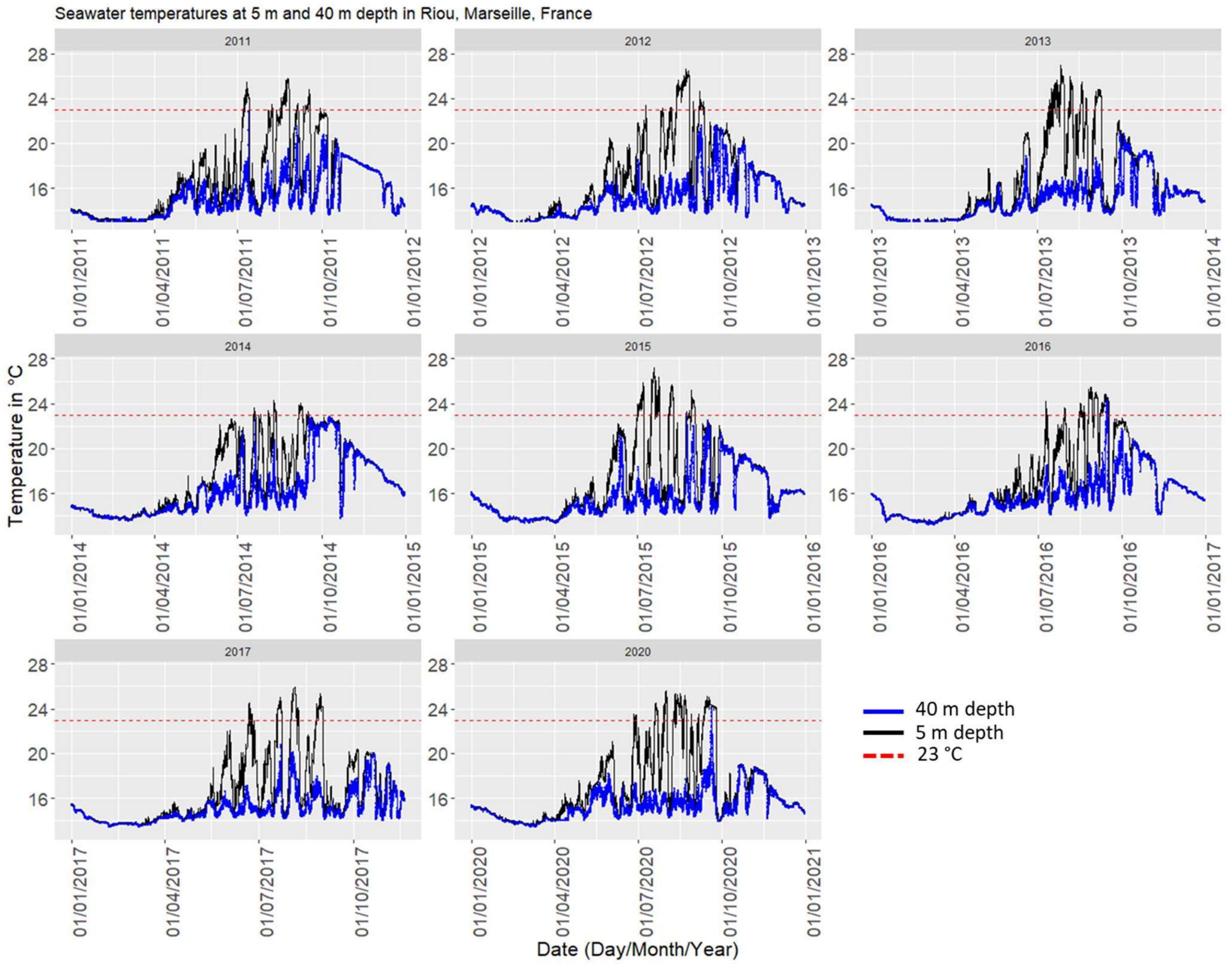

Figure S1: Seawater temperature at 5 m (black) and 40 m (blue) depth from 2011 to 2020 in Riou, Marseille, France. Data obtained from https://t-mednet.org/.

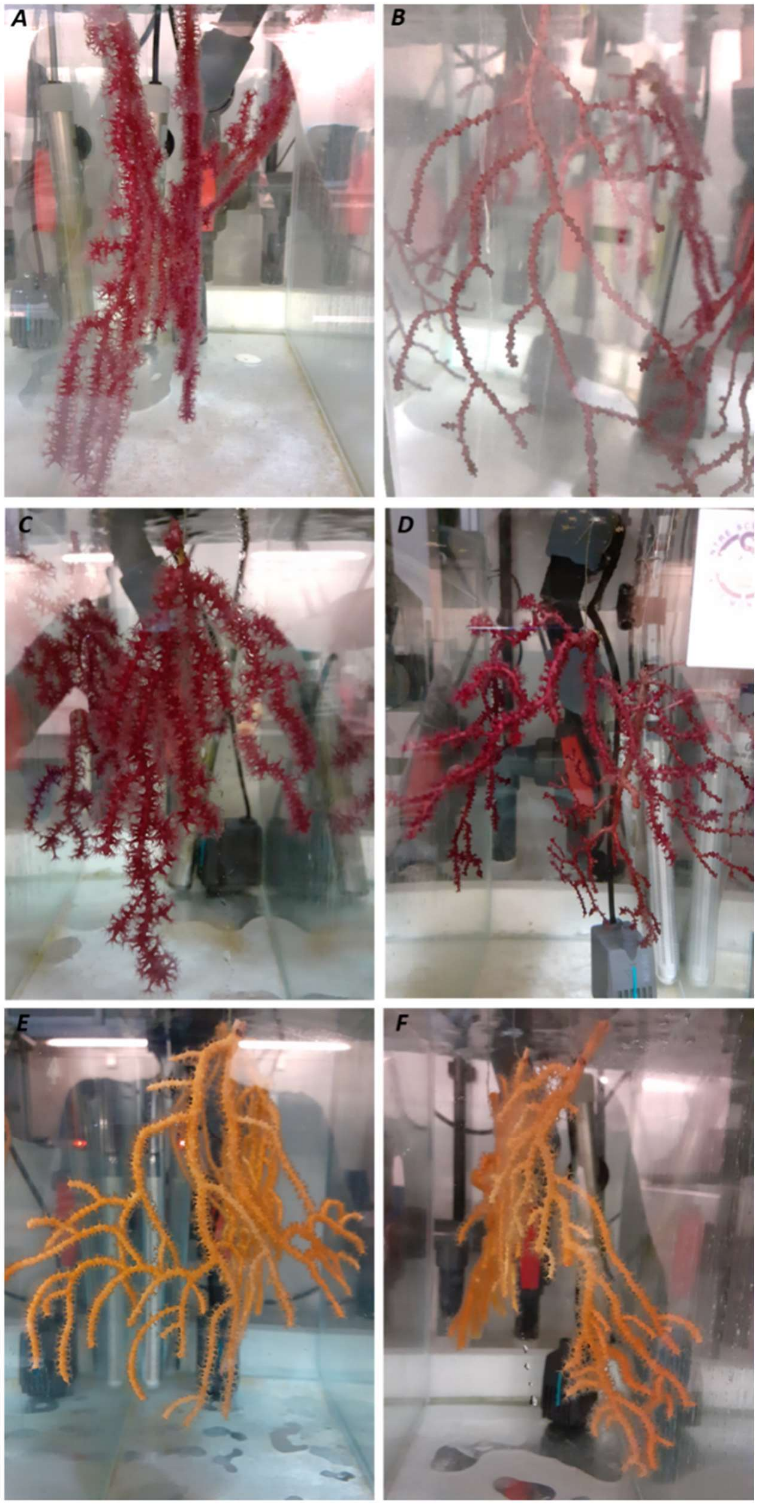

Figure S2: Morphological aspect of gorgonian colonies under both thermal conditions at the end of the experiment. *Paramuricea clavata* colony under control (***A*** and ***C*** for colony 3 and 5, respectively) and stress (***B*** and ***D*** for colony 3 and 5, respectively) condition. *Eunicella cavolini* colony 5 under control (***E***) and stress (***F***) condition.

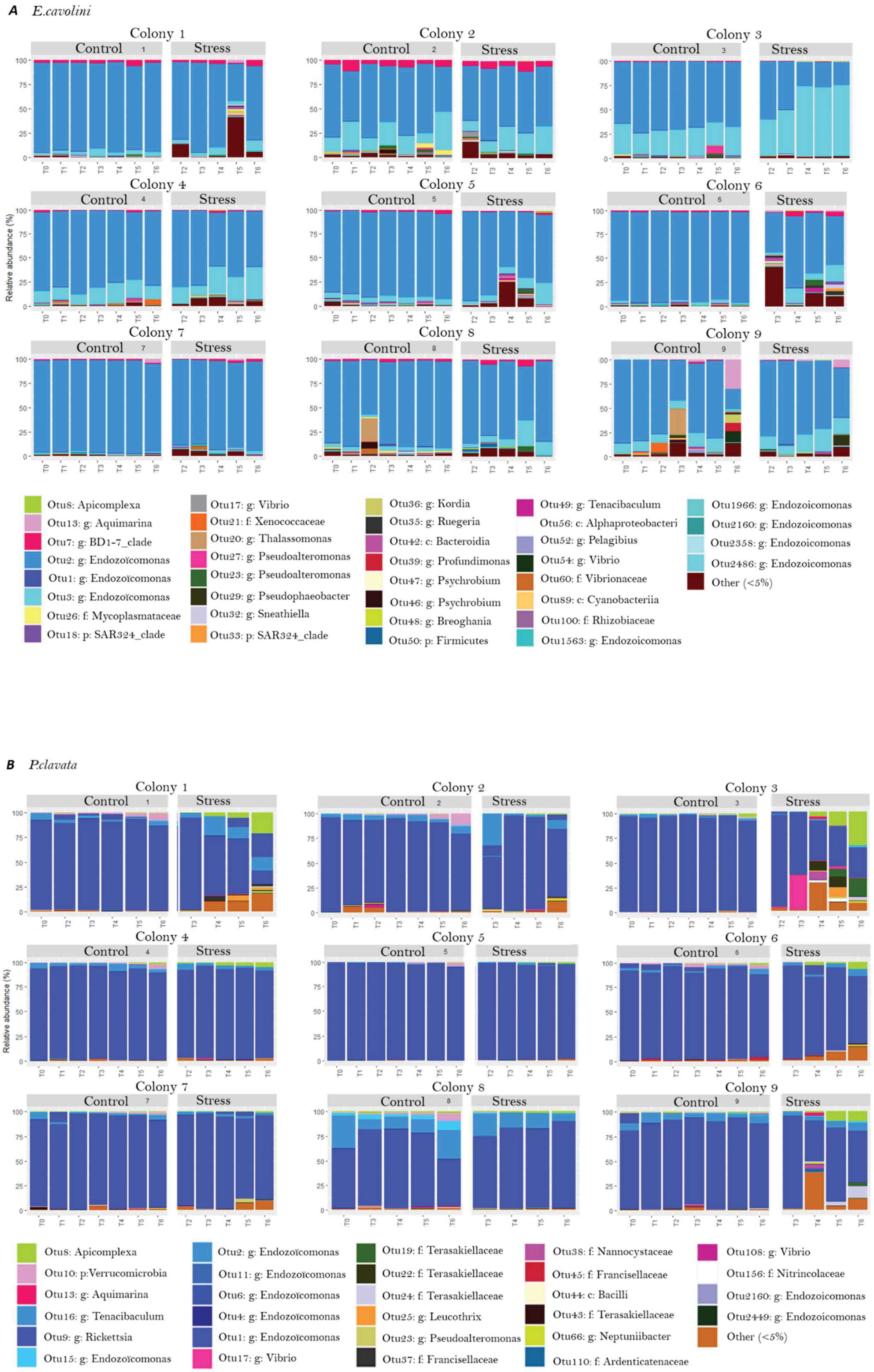

Figure S3: Relative abundance of the most abundant bacterial OTU over time. Relative composition of the most abundant OTUs composing the bacterial community associated with the nine *E. cavolini* (**A**) and *P. clavata* (**B**) colonies under the control and stress conditions over time. The number of samples differs between colonies due to the removal of the samples for which sequencing failed.

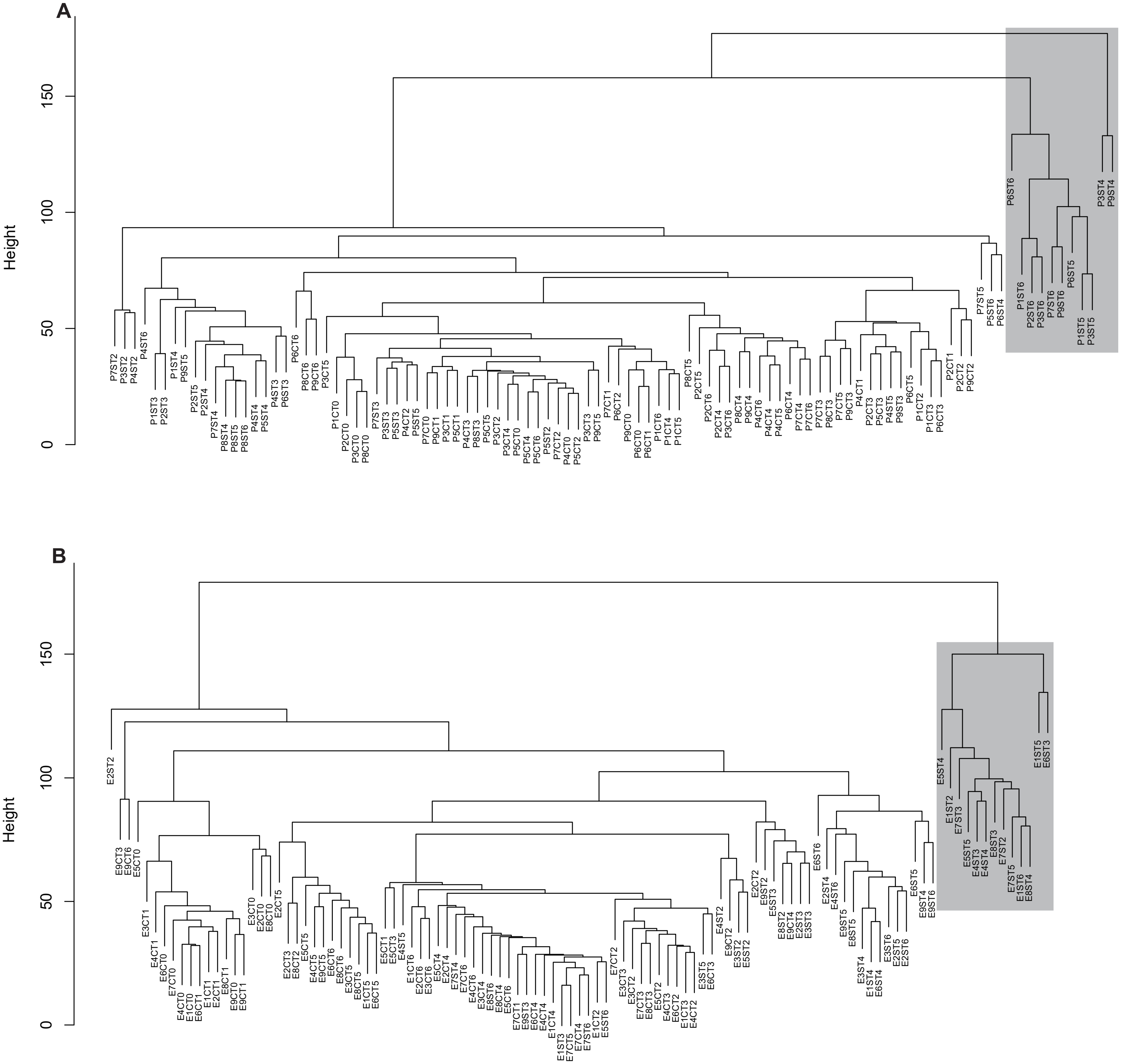

Figure S4: Distribution of the samples based on the bacterial community structure. Hierarchical cluster analysis (WardD2 method) on the Aitchison distance matrix based on the structure of the bacterial community (OTU level) associated with (**A**) *P. clavata* and (**B**) *E. cavolini*. Colonies withat least two samples from different time points (T4, T5, T6) falling outside of the main cluster of the clustering tree were assigned to Group A, and the remaining samples to Group B.

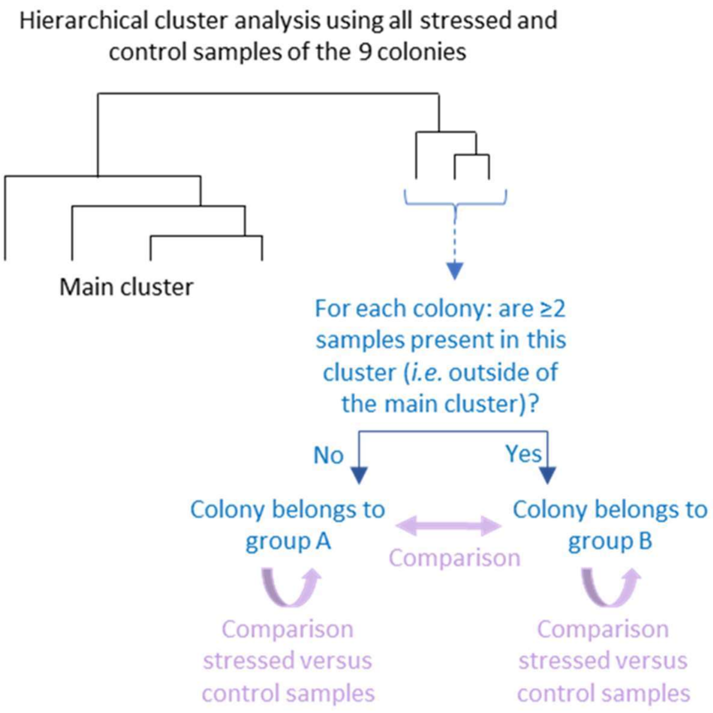

Figure S5: Diagram showing how colonies were assigned to group A or B, and how beta diversity comparisons were made.

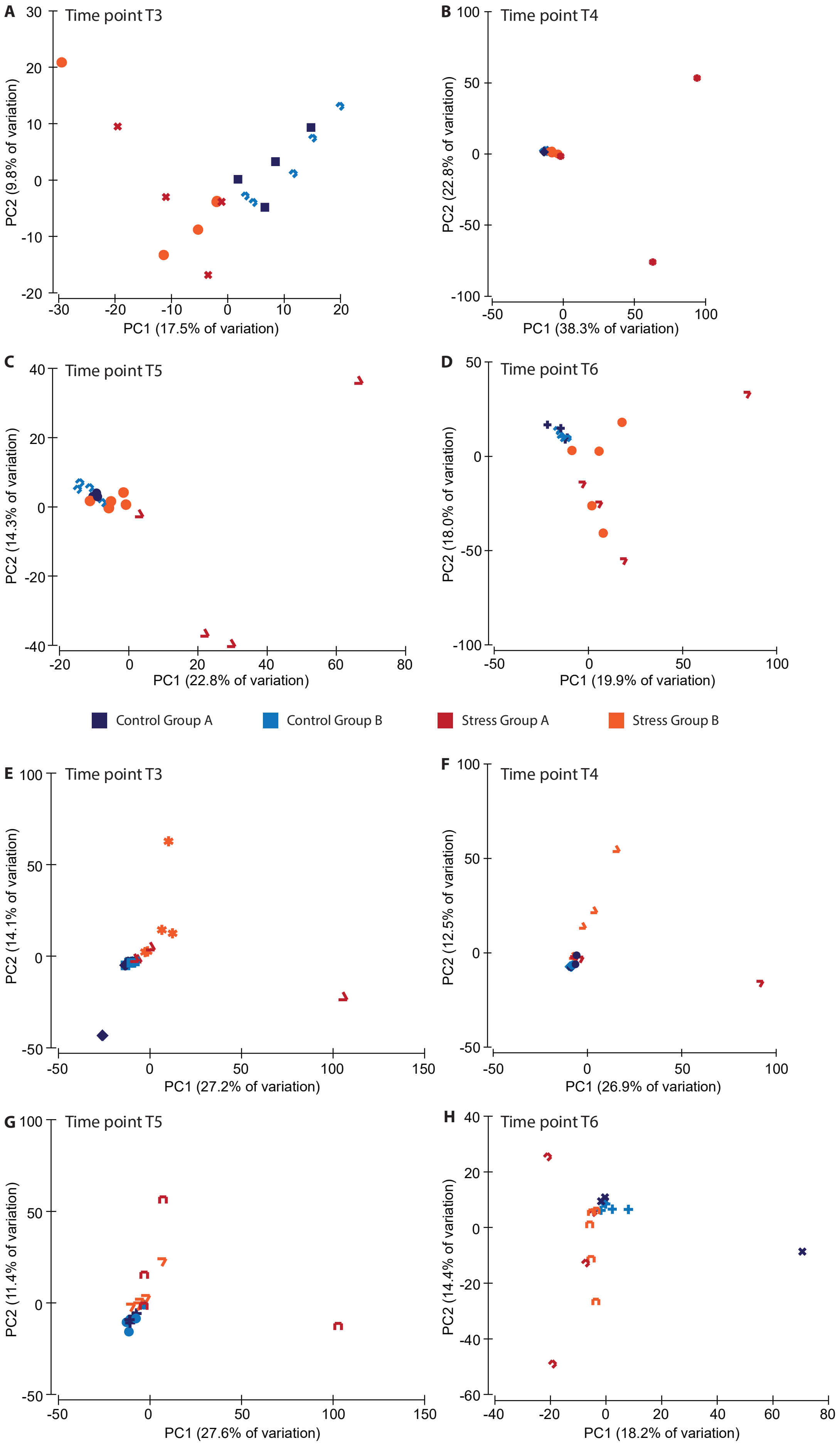

Figure S6: Beta diversity at different time points. Principal Component Analysis (PCA) on centered log-ratio transformed count data was performed at each time point where thermal condition was shown to have an impact on beta diversity of the bacterial communities (T3 - T6) associated with *P. clavata* (**A-D**) and *E. cavolini* (**E-H**). Colors indicated the different thermal conditions (control and stress) and groups (A and B) identified.

A

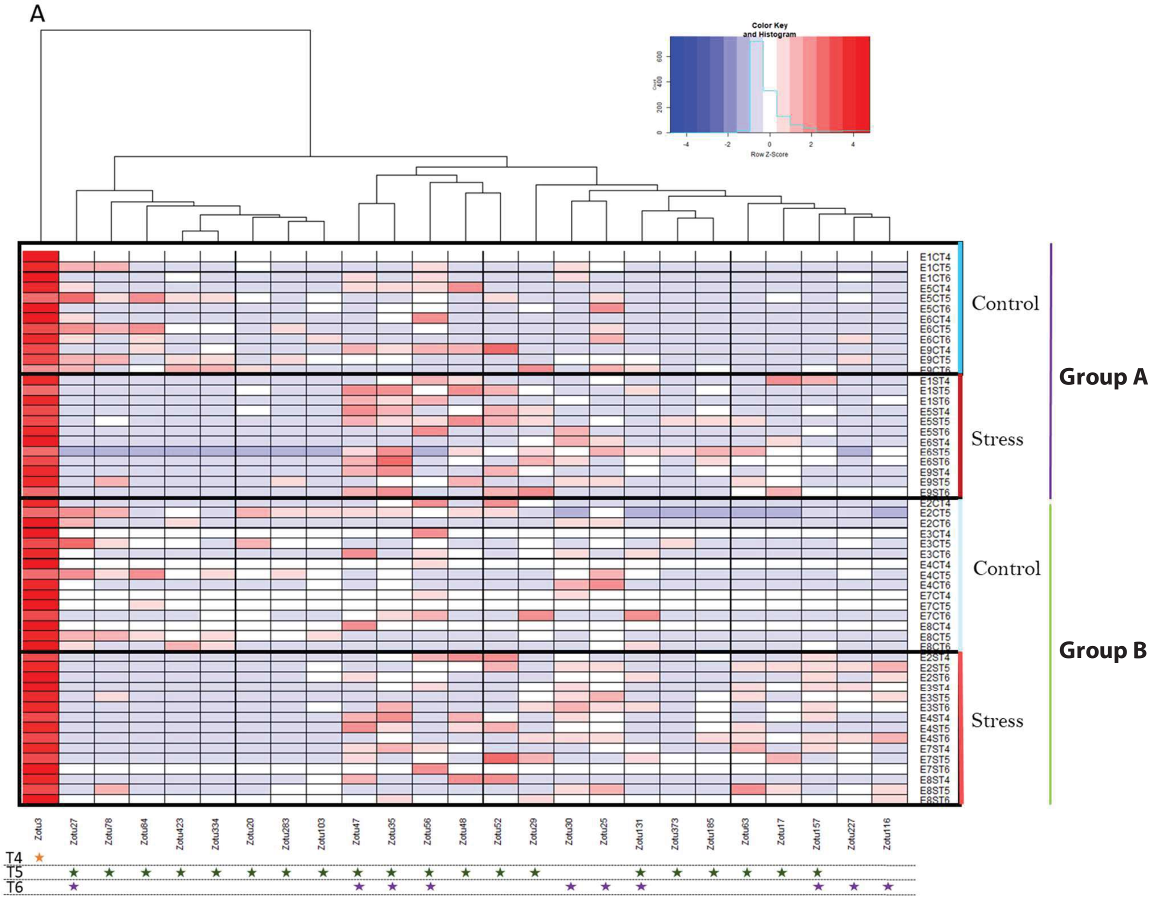

B

**Zotu**	**Taxonomy**
Zotu0003	p:Proteobacteria c:Gammaproteobacteria o:Oceanospirillales f:Endozoicomonadaceae g:*Endozoicomonas*
Zotu0017	p:Proteobacteria c:Gammaproteobacteria o:Vibrionales f:Vibrionaceae g:*Vibrio*
Zotu0020	p:Proteobacteria c:Gammaproteobacteria o:Alteromonadales f:Colwelliaceae g:*Thalassomonas*
Zotu0025	p:Proteobacteria c:Gammaproteobacteria o:Thiotrichales f:Thiotrichaceae g:*Leucothrix*
Zotu0027	p:Proteobacteria c:Gammaproteobacteria o:Alteromonadales f:Pseudoalteromonadaceae g:*Pseudoalteromonas*
Zotu0029	p:Proteobacteria c:Alphaproteobacteria o:Rhodobacterales f:Rhodobacteraceae g:*Pseudophaeobacter*
Zotu0030	p:Cyanobacteria c:Cyanobacteriia o:Chloroplast f:uncultured organism
Zotu0035	p:Proteobacteria c:Alphaproteobacteria o:Rhodobacterales f:Rhodobacteraceae g:*Ruegeria*
Zotu0047	p:Proteobacteria c:Alphaproteobacteria o:Rhizobiales f:Stappiaceae g:*Labrenzia*
Zotu0048	p:Proteobacteria c:Alphaproteobacteria o:Rhizobiales f:Stappiaceae g:*Breoghania*
Zotu0052	p:Proteobacteria c:Alphaproteobacteria o:Kiloniellales f:Kiloniellaceae g:*Pelagibius*
Zotu0056	p:Proteobacteria c:Alphaproteobacteria
Zotu0063	p:Proteobacteria c:Gammaproteobacteria o:Alteromonadales f:Colwelliaceae g:*Thalassotalea*
Zotu0078	p:Proteobacteria c:Gammaproteobacteria o:Burkholderiales f:T34
Zotu0084	p:Proteobacteria c:Gammaproteobacteria o:Alteromonadales f:Colwelliaceae g:*Thalassotalea*
Zotu0103	p:Proteobacteria c:Gammaproteobacteria o:Alteromonadales f:Marinobacteraceae g:*Marinobacter*
Zotu0116	p:Bacteroidota c:Bacteroidia o:Cytophagales f:Microscillaceae g:*Microscilla*
Zotu0131	p:Proteobacteria c:Alphaproteobacteria o:Rhizobiales f:Devosiaceae g:*Maritalea*
Zotu0157	p:Proteobacteria c:Alphaproteobacteria o:Rhizobiales f:Methyloligellaceae g:uncultured
Zotu0185	p:Proteobacteria c:Alphaproteobacteria o:Parvibaculales f:Parvibaculaceae g:Candidatus_*Phaeomarinobacter*
Zotu0227	p:Proteobacteria c:Gammaproteobacteria o:Alteromonadales f:Idiomarinaceae g:*Idiomarina*
Zotu0283	p:Bacteroidotac:Bacteroidia o:Flavobacteriales f:Weeksellaceae g:*Chryseobacterium*
Zotu0334	p:Proteobacteria c:Gammaproteobacteria o:Oceanospirillales f:Nitrincolaceae g:*Profundimonas*
Zotu0373	p:Proteobacteria c:Gammaproteobacteria o:Alteromonadales f:Colwelliaceae g:*Thalassotalea*
Zotu0423	p:Proteobacteria c:Gammaproteobacteria o:Oceanospirillales f:Nitrincolaceae g:*Profundimonas*

Figure S7: Heatmaps of the OTUs whose relative abundance significantly changed under thermal stress. (**A**) Log10-transformed relative abundance of the OTUs that changed under stress within the microbiota of *E. cavolini* in at least one of the two groups (i.e. A and B groups) (ANCOMbc results). The colored stars indicate at which time point the differential abundance was observed. The taxonomy of the differentially abundant OTUs are presented in (**B**).

A

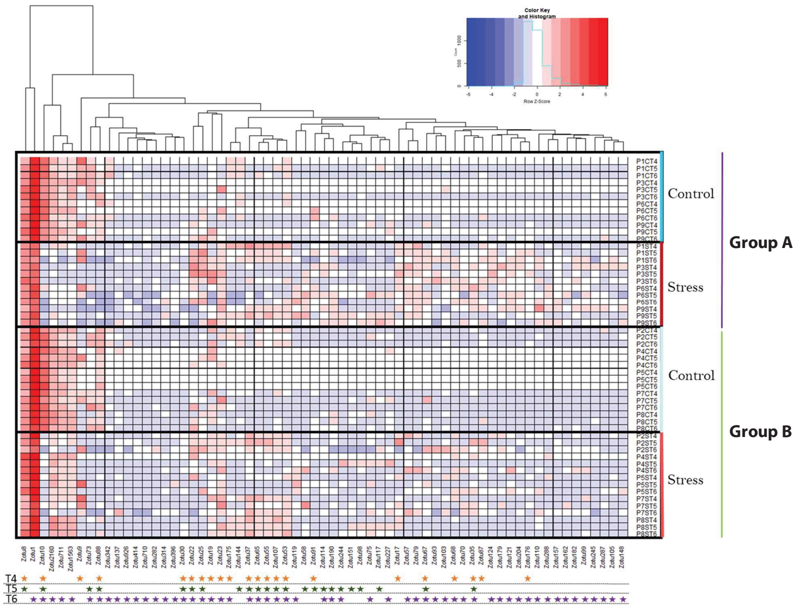

B

Zotu	Taxonomy
Zotu0001	p:Proteobacteria c:Gammaproteobacteria o:Oceanospirillales f:Endozoicomonadaceae g:Endozoicomonas
Zotu0008	p:Cyanobacteria c:Cyanobacteriia o:Chloroplast
Zotu0009	p:Proteobacteria c:Alphaproteobacteria o:Rickettsiales f:Rickettsiaceae g:*Rickettsia*
Zotu0010	p:Verrucomicrobiota
Zotu0017	p:Proteobacteria c:Gammaproteobacteria o:Vibrionales f:Vibrionaceae g:*Vibrio*
Zotu0019	p:Proteobacteria c:Alphaproteobacteria o:Rhodospirillales f:Terasakiellaceae g:uncultured
Zotu0022	p:Proteobacteria c:Alphaproteobacteria o:Rhodospirillales f:Terasakiellaceae g:uncultured
Zotu0023	p:Proteobacteria c:Gammaproteobacteria o:Alteromonadales f:Pseudoalteromonadaceae g:*Pseudoalteromonas*
Zotu0025	p:Proteobacteria c:Gammaproteobacteria o:Thiotrichales f:Thiotrichaceae g:*Leucothrix*
Zotu0030	p:Cyanobacteria c:Cyanobacteriia o:Chloroplast f:uncultured organism
Zotu0035	p:Proteobacteria c:Alphaproteobacteria o:Rhodobacterales f:Rhodobacteraceae g:*Ruegeria*
Zotu0037	p:Proteobacteria c:Gammaproteobacteria o:Francisellales f:Francisellaceae g:uncultured
Zotu0053	p:Proteobacteria c:Alphaproteobacteria o:Rickettsiales f:Fokiniaceae g:MD3-55
Zotu0055	p:Proteobacteria c:Gammaproteobacteria
Zotu0057	p:Bacteroidotac:Bacteroidia o:Flavobacteriales f:Flavobacteriaceae g:*Tenacibaculum*
Zotu0058	p:Proteobacteria c:Gammaproteobacteria o:Oceanospirillales f:Saccharospirillaceae g:*Litoribacillus*
Zotu0065	p:Proteobacteria c:Alphaproteobacteria o:Rickettsiales f:Fokiniaceae g:MD3-55
Zotu0067	p:Proteobacteria c:Gammaproteobacteria o:Alteromonadales f:Alteromonadaceae g:*Alteromonas*
Zotu0068	p:Proteobacteria c:Gammaproteobacteria
Zotu0070	p:Proteobacteria c:Alphaproteobacteria o:Kiloniellales f:Kiloniellaceae g:*Kiloniella*
Zotu0073	p:Bacteroidotac:Bacteroidia o:Flavobacteriales f:Flavobacteriaceae g:*Wenyingzhuangia*
Zotu0075	p:Proteobacteria c:Gammaproteobacteria o:Oceanospirillales f:Halomonadaceae
Zotu0079	p:Proteobacteria c:Alphaproteobacteria o:Rhodobacterales f:Rhodobacteraceae g:*Phaeobacter*
Zotu0087	p:Proteobacteria c:Alphaproteobacteria o:Rhizobiales f:Methyloligellaceae g:uncultured
Zotu0088	p:Firmicutes c:Bacilli o:Mycoplasmatales f:Mycoplasmataceae g:*Mycoplasma*
Zotu0091	p:Proteobacteria c:Gammaproteobacteria o:Vibrionales f:Vibrionaceae g:*Vibrio*
Zotu0093	p:Proteobacteria c:Gammaproteobacteria o:Cellvibrionales f:Cellvibrionaceae g:*Halioxenophilus*
Zotu0098	p:Proteobacteria c:Gammaproteobacteria o:Oceanospirillales f:Halomonadaceae g:*Cobetia*
Zotu0099	p:Proteobacteria c:Alphaproteobacteria o:Rhizobiales f:Rhizobiaceae g:*Cohaesibacter*
Zotu0103	p:Proteobacteria c:Gammaproteobacteria o:Alteromonadales f:Marinobacteraceae g:*Marinobacter*
Zotu0105	p:Proteobacteria c:Alphaproteobacteria o:Rhodobacterales f:Rhodobacteraceae g:*Ruegeria*
Zotu0107	p:Proteobacteria c:Gammaproteobacteria o:Francisellales f:Francisellaceae
Zotu0110	p:Chloroflexi c:Anaerolineae o:Ardenticatenales f:Ardenticatenaceae g:uncultured
Zotu0114	p:Proteobacteria c:Gammaproteobacteria o:Oceanospirillales f:Kangiellaceae g:*Aliikangiella*
Zotu0117	p:Proteobacteria c:Gammaproteobacteria o:Oceanospirillales f:Halomonadaceae
Zotu0119	p:Proteobacteria c:Gammaproteobacteria o:Alteromonadales f:Alteromonadaceae g:*Aestuariibacter*
Zotu0121	p:Bacteroidotac:Bacteroidia o:Flavobacteriales f:Flavobacteriaceae g:*Winogradskyella*
Zotu0124	p:Proteobacteria c:Gammaproteobacteria o:Oceanospirillales f:Kangiellaceae g:*Aliikangiella*
Zotu0137	p:Proteobacteria c:Gammaproteobacteria o:Alteromonadales f:Marinobacteraceae g:*Marinobacter*
Zotu0144	p:Proteobacteria c:Gammaproteobacteria o:Francisellales f:Francisellaceae g:[*Caedibacter*]_*taeniospiralis*_group
Zotu0148	p:Chloroflexi c:Anaerolineae o:Ardenticatenales f:Ardenticatenaceae g:uncultured
Zotu0151	p:Proteobacteria c:Gammaproteobacteria o:Alteromonadales f:Idiomarinaceae g:*Idiomarina*
Zotu0157	p:Proteobacteria c:Alphaproteobacteria o:Rhizobiales f:Methyloligellaceae g:uncultured
Zotu0162	p:Proteobacteria c:Gammaproteobacteria o:Cellvibrionales f:Cellvibrionaceae
Zotu0175	p:Proteobacteria c:Gammaproteobacteria o:Francisellales f:Francisellaceae g:[*Caedibacter*]_*taeniospiralis*_group
Zotu0176	p:Proteobacteria c:Alphaproteobacteria o:Rhodobacterales f:Rhodobacteraceae g:uncultured
Zotu0179	p:Proteobacteria c:Gammaproteobacteria
Zotu0182	p:Proteobacteria c:Gammaproteobacteria o:Cellvibrionales f:Cellvibrionaceae g:*Pseudoteredinibacter*
Zotu0190	p:Proteobacteria c:Gammaproteobacteria o:Oceanospirillales f:Oleiphilaceae g:*Oleiphilus*
Zotu0204	p:Proteobacteria c:Alphaproteobacteria o:Rhodobacterales f:Rhodobacteraceae g:uncultured
Zotu0227	p:Proteobacteria c:Gammaproteobacteria o:Alteromonadales f:Idiomarinaceae g:*Idiomarina*
Zotu0244	p:Proteobacteria c:Gammaproteobacteria o:Alteromonadales f:Idiomarinaceae g:*Idiomarina*
Zotu0245	p:Proteobacteria c:Gammaproteobacteria o:Ectothiorhodospirales f:Ectothiorhodospiraceae g:uncultured
Zotu0282	p:Proteobacteria c:Gammaproteobacteria o:Oceanospirillales f:Alcanivoracaceae1 g:*Alcanivorax*
Zotu0287	p:Proteobacteria c:Alphaproteobacteria o:Rhodobacterales f:Rhodobacteraceae
Zotu0288	p:Myxococcota c:Polyangia o:Nannocystales f:Nannocystaceae g:uncultured
Zotu0314	p:Proteobacteria c:Gammaproteobacteria
Zotu0342	p:Firmicutes c:Bacilli
Zotu0396	p:Proteobacteria c:Alphaproteobacteria o:Sphingomonadales f:Sphingomonadaceae g:*Erythrobacter*
Zotu0414	p:Proteobacteria c:Gammaproteobacteria o:Enterobacterales f:Enterobacteriaceae g:*Escherichia-Shigella*
Zotu0710	p:Myxococcota c:Polyangia o:Haliangiales f:Haliangiaceae g:*Haliangium*
Zotu0711	p:Proteobacteria c:Gammaproteobacteria o:Oceanospirillales f:Endozoicomonadaceae g:*Endozoicomonas*
Zotu0926	p:Proteobacteria c:Alphaproteobacteria o:Rickettsiales f:AB1
Zotu1563	p:Proteobacteria c:Gammaproteobacteria o:Oceanospirillales f:Endozoicomonadaceae g:*Endozoicomonas*
Zotu2160	p:Proteobacteria c:Gammaproteobacteria o:Oceanospirillales f:Endozoicomonadaceae g:*Endozoicomonas*

Figure S8: Heatmaps of the OTUs whose relative abundance significantly changed under thermal stress. (**A**) Log10-transformed relative abundance of the OTUs that changed under stress within the microbiota of *P. clavata* in at least one of the two groups (i.e. A and B groups) (ANCOMbc results). The colored stars indicate at which time point the differential abundance was observed. The taxonomy of the differentially abundant OTUs are presented in (**B**).
